# Depressed chemical weathering inhibited climate stabilization after the end-Permian biocrisis

**DOI:** 10.1126/sciadv.aee6350

**Published:** 2026-10-28

**Authors:** Guang-Yi Wei, Feifei Zhang, Hua Zhang, Yi-Bo Lin, Yi-Sheng Yin, Mohammad N. Gorgij, Sakineh Arefifard, Thomas J. Algeo, Philip A. E. Pogge von Strandmann, Benjamin J. W. Mills, Shu-zhong Shen

**Affiliations:** ^1^ State Key Laboratory of Critical Earth Material Cycling and Mineral Deposits, School of Earth Sciences and Engineering, and Frontiers Science Center for Critical Earth Material Cycling, Nanjing University, 163 Xianlin Avenue, Nanjing 210023, China.; ^2^ International Joint Laboratory for Life Evolution and Carbon Cycling, Nanjing University, Nanjing 210023, China.; ^3^ State Key Laboratory of Paleobiology and Stratigraphy, Nanjing Institute of Geology and Paleontology and Center for Excellence in Life and Paleoenvironment, Chinese Academy of Sciences, 39 East Beijing Road, Nanjing 210008, China.; ^4^ University of Sistan and Baluchestan, Zahedan, Iran.; ^5^ Department of Geology, Lorestan University, Khorramabad, Lorestan 68151-44316, Iran.; ^6^ Department of Geosciences, University of Cincinnati, Cincinnati, OH 45221-0013, USA.; ^7^ State Key Laboratory of Geomicrobiology and Environmental Changes (GMEC), China University of Geosciences-Wuhan, Wuhan 430074, China.; ^8^ State Key Laboratory of Oil and Gas Reservoir Geology and Exploitation, Institute of Sedimentary Geology, Chengdu University of Technology, Chengdu 610059, China.; ^9^ MIGHTY, Institute of Geosciences, Johannes Gutenberg University, 55122 Mainz, Germany.; ^10^ LOGIC, Department of Earth Sciences, University College London, London, UK.; ^11^ School of Earth and Environment, University of Leeds, Leeds, UK.

## Abstract

The Permian-Triassic transition (∼252 million years ago) marks the most severe mass extinction in Earth’s history, followed by a protracted interval of environmental instability and delayed biological recovery. Although elevated atmospheric carbon dioxide (CO_2_) from extensive volcanism is widely invoked, the role of continental weathering feedbacks in regulating global climate has not been effectively constrained. Here, we present high-resolution lithium isotope records from marine carbonates and terrestrial deposits through the Permian-Triassic transition. Combined with an Earth-system model, our data reveal notable decreases in global continental weatherability following the end-Permian mass extinction, inferred to associations with widespread deforestation (extinction of deep-rooted plants) and the collapse of terrestrial ecosystem. This diminished weathering response to volcanic carbon emissions likely contributed to prolonged high atmospheric CO_2_ and global temperature during the Early Triassic. The sustained greenhouse conditions, in turn, inhibited contemporaneous marine and terrestrial ecosystem recoveries. Our findings highlight the importance of terrestrial ecosystem in modulating global carbon cycle stability, and its effects on Earth’s habitability.

## INTRODUCTION

The end-Permian mass extinction (EPME; ∼252 Ma) witnessed the most severe biodiversity crisis of the Phanerozoic, with substantial losses of marine and terrestrial species (*1*, *2*). This mass extinction event was followed by a protracted interval of biological recovery, limited ecosystem complexity, and major biogeochemical perturbations during the Early Triassic (∼252 to 247 Ma) (*3*–*5*). The deterioration of global surface environments (e.g., extreme climate, oceanic anoxia, and acidification) is regarded as a major cause of the EPME and the subsequent delay in Earth-system recovery (*6*–*9*). It is known that the global environmental crisis was primarily triggered by large igneous province volcanism beginning in the latest Permian (*10*, *11*), resulting in massive emissions of greenhouse gases (CO_2_ and CH_4_) and toxins (e.g., halogens) and large negative δ^13^C excursions during the Permian-Triassic (P-Tr) transition (*12*–*15*).

Although the effects of volcanism have been widely inferred, multiple scenarios have been proposed for the causal mechanisms of the extinction and marine environmental deterioration, as well asthe delayed climate stabilization and protracted biological recovery during the Early Triassic (*16*, *17*). The P-Tr transition is marked by rapid increases in atmospheric CO_2_ and surface temperatures, persisting for more than 1 Myr into the Early Triassic (*12*–*14*), although the initial pulse of volcanic carbon emissions associated with the EPME likely lasted only ∼100 kyr (*10*, *18*). Compared to other hyperthermal events during the Phanerozoic, for instance, Mesozoic Oceanic Anoxic Events (OAEs) and Paleocene-Eocene Thermal Maximum (PETM), the duration of global warming during the P-Tr transition was longer, with high atmospheric CO_2_ levels and a hothouse climate persisting through the Early Triassic (*19*). At timescales of >10^6^ years, continental silicate weathering plays a critical role in regulating atmospheric CO_2_ and global climate, acting as Earth’s geological thermostat (*20*–*22*). Increases in atmospheric CO_2_ and temperature tend to enhance continental silicate weathering, thus consuming CO_2_ and facilitating climate stabilization following hyperthermal events of the Phanerozoic (*19*, *23*). However, the silicate weathering feedback and its effective impact on global climate during the P-Tr transition still remain in dispute based on current research (*23*). A range of proxies, including sedimentological records (*24*), the chemical index of alteration (CIA) (*25*), radiogenic strontium isotopes (^87^Sr/^86^Sr) and lithium isotopes (δ^7^Li) (*26*, *27*), have been used to reconstruct continental weathering across the P-Tr transition. However, the magnitude and climatic impact of silicate weathering feedbacks during this interval remain poorly known. This uncertainty reflects the scarcity of continuous, high-resolution records and the lack of quantitative constraints on chemical weathering rates at a global scale. In addition, emerging evidence for strong regional heterogeneity in weathering responses further complicates efforts to link continental weathering to global warming and carbon cycle dynamics during this critical transition.

Here, we conduct high-resolution δ^7^Li analyses in both marine carbonate and terrestrial siliciclastic successions in multiple regions globally to trace changes in continental silicate weathering during the P-Tr transition. Combining these δ^7^Li data with an Earth-system model, we attribute persistently high atmospheric CO_2_ and temperature to depressed operation of the continental silicate weathering feedback, potentially linked to deforestation (i.e., extinction of deep-rooted plants) and terrestrial ecosystem collapse during the EPME. Concurrently, decreased weathering intensity and reduced clay formation is inferred to have amplified the consumption of dissolved O_2_ in seawater by decreasing organic carbon burial efficiency, contributing to prolonged marine anoxia and delayed Earth-system recovery in the Early Triassic.

## RESULTS AND DISCUSSION

### Carbonate δ^7^Li as a record of global silicate weathering intensity during the P-Tr transition

Four carbonate sections from South China, Iran, and southern Austria were used to generate continuous records of seawater Li isotopic compositions from the latest Permian to earliest Triassic (Fig. 1). Despite their wide geographic separation and varying depositional facies, these four carbonate sections exhibit consistent δ^7^Li patterns, marked by a decrease from ∼+19‰ in the uppermost Permian to ∼+9‰ within the P-Tr boundary interval, and back to ∼+15‰ in the lowermost Triassic (Fig. 1B). The molar ratios of Al/(Ca + Mg) in the study samples were examined to evaluate the detrital contamination during sample leaching (fig. S1). By using a sequential leaching approach with 1 M acetic acid, we find that most of the leached carbonate fractions are marked by Al/(Ca + Mg) ratios below the recommended threshold value of 0.8 mmol/mol for ancient carbonates (*28*). Even using a more conservative threshold value of 0.45 (*29*), most carbonates above this value are only from the Zal section. Overall, no substantial correlations among the δ^7^Li, Li/(Ca + Mg), and Al/(Ca + Mg) values are observed for all the study sections, except for a weak correlation between δ^7^Li and Al/(Ca + Mg) at the Zal section (fig. S1). Thus, we suggest that potential detrital effects on carbonate δ^7^Li may have been limited to the Zal section and that the consistent δ^7^Li records among the four sections reliably reflect the isotopic compositions of carbonates.

**Fig. 1.**
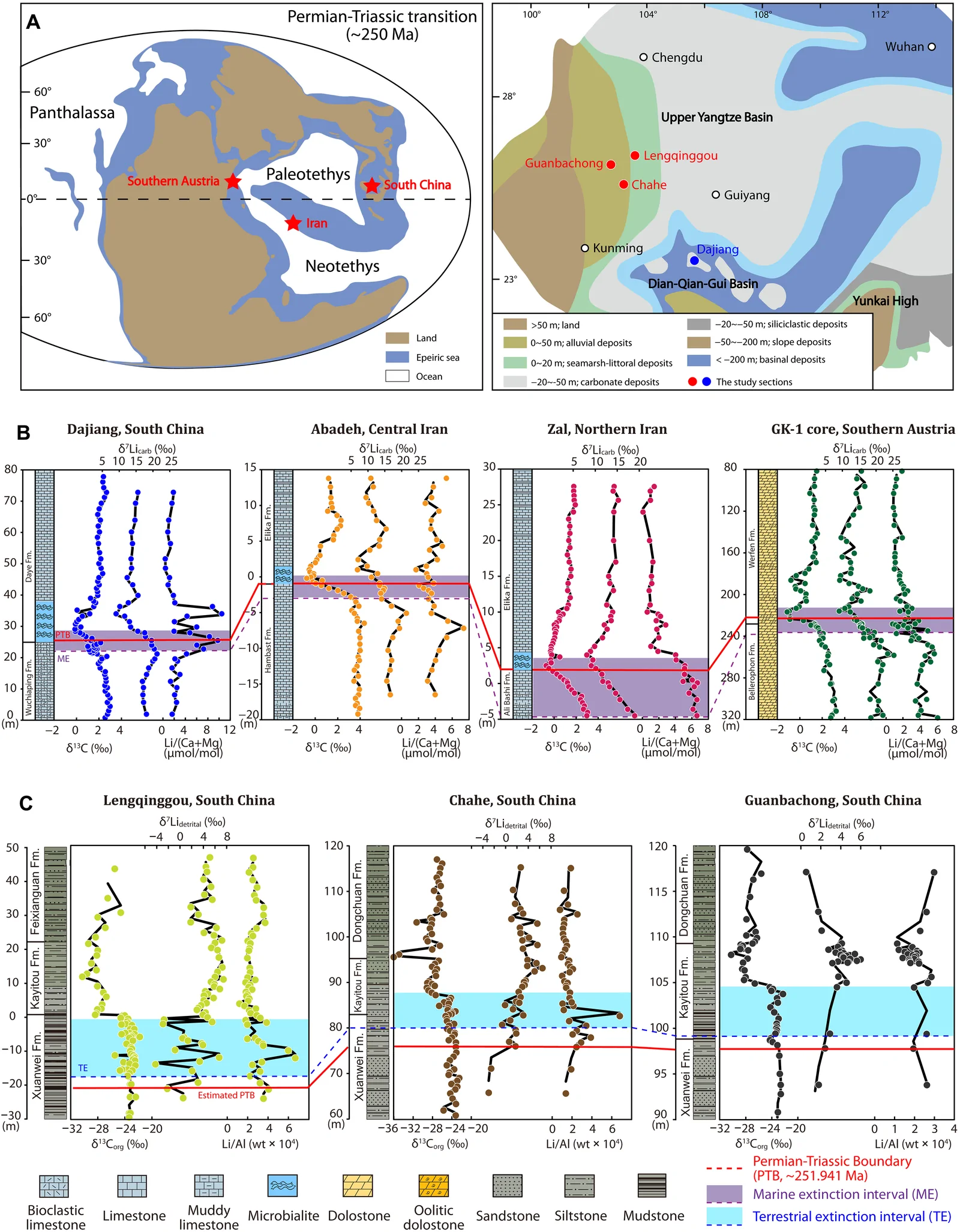
Paleogeography and locations of the study sections and geochemical profiles of marine carbonates and terrestrial successions during the P-Tr transition. (**A**) Paleogeographic maps of the study sections. Reconstruction of global paleogeography during the P-Tr transition in (A) is adapted from a published reconstruction model (*91*). The paleographic map of South China in (A) is modified based on (*40*). (**B**) Chemostratigraphic profiles of carbonate sections. (**C**) Chemostratigraphic profiles of terrestrial sections. The time framework for marine and terrestrial extinction intervals and P-Tr boundary in (B) and (C) is modified based on (*37*, *38*, *40*).

Although seawater pH and temperature do not notably influence Li isotope fractionations between inorganic carbonate and seawater (*30*, *31*), the δ^7^Li values of shallow-marine carbonates are closely linked to carbonate mineralogy and early diagenesis. Substantial isotopic fractionations occur during the precipitation of primary aragonite or high-Mg calcite, but mute fractionations are observed in diagenetic low-Mg calcite and dolomite under seawater-buffered conditions (*29*, *32*, *33*). Additionally, meteoric diagenetic carbonates are typically characterized by much lower δ^7^Li relative to coeval seawater, reflecting overprinting by freshwater with substantially low δ^7^Li signatures during water-rock interaction. Although subaerial exposure surfaces and associated depositional hiatuses have been reported in carbonate successions from South China and Iran (*34*, *35*), these features are stratigraphically confined to a very short interval in the lower part of the conodont *Clarkina meishanensis* zone slightly below the P-Tr boundary (fig. S1) (*35*). Particularly, such discontinuities are not vertically persistent through the study carbonate successions and, therefore, do not constitute evidence for sustained meteoric diagenesis during the P-Tr transition. This stratigraphic framework contrasts with well-developed meteoric diagenetic zones in Pleistocene shallow-water carbonates from the Bahamas and South China Sea, where prolonged subaerial exposure produced pervasive and vertically extensive alteration profiles (*36*). Geochemical systematics further preclude a dominant meteoric diagenetic overprint in the study successions. For all sections, δ^13^C and δ^7^Li records do not exhibit systematic covariance with δ^18^O (fig. S2). Instead, δ^18^O values display a progressive decrease from the uppermost Permian into the lowest Triassic, consistent with temperature-dependent fractionation under rising seawater temperatures rather than freshwater infiltration. Moreover, δ^13^C and δ^18^O show no statistically notable correlations in the majority of sections (fig. S3A), indicating the absence of coupled diagenetic resetting. The only exception is the GK-1 drill core, where δ^13^C and δ^18^O covariation is observed. However, this relationship is restricted to dolostone intervals and is more plausibly attributed to diagenetic dolomitization, which can induce coupled shifts in δ^13^C and δ^18^O values through recrystallization processes, rather than meteoric alteration. In particular, exclusion of samples from the uppermost Permian interval, characterized by elevated δ^13^C and δ^18^O values, eliminates this covariation, yielding no resolvable δ^13^C and δ^18^O relationship across the P-Tr boundary. An analogous pattern is observed for δ^7^Li and δ^18^O systematics—only GK-1 dolostones show an appreciable correlation (fig. S3B), which likewise disappears upon removal of uppermost Permian samples. Together, these observations demonstrate that the study sections did not undergo pervasive meteoric diagenesis and that the pronounced negative δ^7^Li excursions across the P-Tr boundary cannot be explained by freshwater overprinting.

Except for several microbial horizons in the Dajiang and Abadeh sections, most carbonates in this study have molar ratios of Li/(Ca + Mg) (0.08 to 6.7 μmol/mol) and Sr/(Ca + Mg) (0.03 to 2.4 mmol/mol) that are low relative to those of primary aragonite and calcite (∼10 to 60 μmol/mol and ∼3 to 12 mmol/mol, respectively) (fig. S3, C and D), suggesting that the study samples are mainly composed of low-Mg calcites or dolomites that were likely to have experienced marine burial diagenesis under fluid-buffered conditions. Excluding the uppermost Permian dolostones in the GK-1 drillcore with coupled high δ^7^Li and δ^18^O values, lack of substantial correlations of δ^7^Li versus Li/(Ca + Mg), Sr/(Ca + Mg), Mg/Ca, and Mn/(Ca + Mg) can be observed (fig. S3, C to F), indicative of no changes in diagenetic regimes (i.e., fluid-buffered or sediment-buffered) or extents for the carbonates through the P-Tr transition. These considerations suggest that the Li isotope system was affected mainly by fluid-buffered marine diagenesis or dolomitization, implying that δ^7^Li variations in the study samples preserve snapshots of seawater δ^7^Li signals from the latest Permian to the earliest Triassic with limited Li isotopic offsets between carbonate and seawater. On this basis, we adopt an offset of −2‰ between carbonate and seawater for carbonates deposited under seawater-buffered conditions, following empirical constraints from the upper Cenozoic bulk carbonates from the Bahamas and the South China Sea (*29*, *33*). While such offsets may, in principle, vary in ancient systems, multiple independent lines of evidence demonstrate that both depositional and diagenetic conditions remained effectively invariant throughout the studied carbonate profiles. In particular, there is no indication of shifts between fluid- and sediment-buffered diagenesis, nor any overprint from meteoric alteration. Hence, the imposed Li isotope offset cannot distort the observed consistent variations in carbonate δ^7^Li through the P-Tr transition, which necessarily reflect primary changes in seawater δ^7^Li signatures. To better compare temporal changes in climate and marine environment during the P-Tr transition, we integrated records of carbonate δ^13^C, carbonate and terrestrial δ^7^Li, and other key events from both marine and terrestrial sections in a geochronological framework, based on zircon U-Pb dating (*2*, *37*, *38*), as well as biostratigraphic and carbon isotope chemostratigraphic correlations (Fig. 2) (*39*, *40*). On a global scale, carbonate δ^7^Li records in widely separated regions from South China, Iran, and Alps in southern Austria yield similar values and secular variation patterns, documenting an abrupt decrease of ∼10‰ in global seawater δ^7^Li coincident with the negative inorganic δ^13^C excursion marking the P-Tr transition (Fig. 2, C and D).

**Fig. 2.**
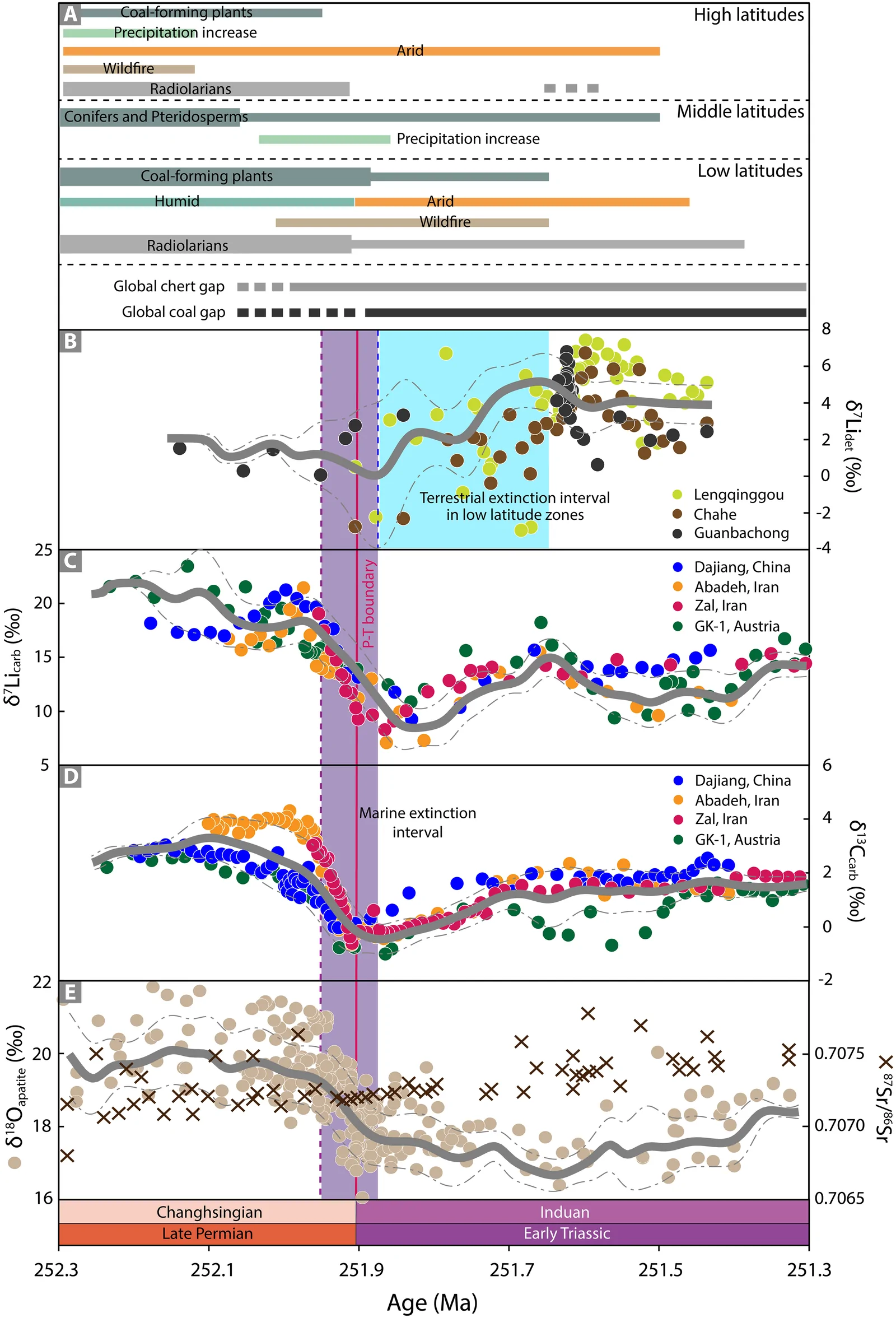
Marine and terrestrial δ^7^Li records and key geological-biological events through the P-Tr transition. (**A**) Key geological-biological events. (**B**) Terrestrial detrital δ^7^Li records. (**C**) Marine carbonate δ^7^Li records. (**D**) Marine carbonate δ^13^C records. (**E**) Conodont apatite δ^18^O and ^87^Sr/^86^Sr records. The temporal framework for marine and terrestrial extinction intervals and key events through the P-Tr transition are modified based on (*9*, *37*, *38*). The gray curves in (B) to (E) are smoothing fits for the isotopic records with 1σ error.

Global seawater δ^7^Li is determined by the oceanic Li isotope mass balance through time, which is regulated by the Li fluxes and isotopic compositions of the sources (i.e., river waters and high-temperature hydrothermal fluids) and sinks (i.e., marine authigenic clays and oceanic crust alteration). On short timescales (<1 Myr), the Li isotopic compositions and fluxes of hydrothermal fluids and oceanic crustal alteration do not notably vary due to limited isotopic fractionations at high temperature and low mid-ocean ridge spreading rates (*41*). Previous studies attributed prolonged low seawater δ^7^Li in the Early Triassic to extensive marine authigenic clay formation (i.e., enhanced marine reverse weathering) and decreased isotopic fractionation as a result of increased marine Si concentrations associated with the extinction of siliceous planktonic species (*42*, *43*). However, abrupt (∼0.1 Myr) decreases in seawater δ^7^Li at the P-Tr boundary cannot have been driven solely by enhanced reverse weathering rates when considering the evolution of the seawater Si inventory. The demise of chert deposition, previously interpreted as evidence for the extinction of siliceous planktonic species, likely commenced well before the P-Tr boundary (*44*). Additionally, recent findings of radiolarians and sponge spicules in some mid- and low-paleolatitude shelf settings provide evidence that decline of biosiliceous productivity may have been delayed through the Early Triassic, precluding rapid increases in marine dissolved Si concentrations and reverse weathering rates during the P-Tr transition (*45*, *46*). Regardless of the pattern of change in the marine Si inventory, low-latitudinal warming of 8° to 10°C during the EPME (*17*) also reduced Li isotopic fractionations during Li removal from seawater, with a temperature dependence of −0.25‰/°C during oceanic crust alteration and marine authigenic clay formation (*47*, *48*). Hence, we propose that changes in Li isotopic fractionation between seawater and Li sinks likely drove a 2.0 to 2.5‰ decrease in seawater δ^7^Li during the P-Tr transition. Together, the above observations suggest that changes in silicate weathering were responsible for abrupt decreases of ∼10‰ in global seawater δ^7^Li during the P-Tr transition, although enhanced reverse weathering may have also facilitated the persistence of relatively low seawater δ^7^Li through the Early Triassic (*42*).

Based on the Li isotope systematics of modern river systems (*49*, *50*), weathering intensity (*W*/*D*, defined as the ratio of chemical weathering rate to total denudation rate), Li fluxes, and the Li isotopic compositions of river water and weathering products are closely interrelated at a global scale. Low *W*/*D* is characterized by low δ^7^Li and high Li flux owing to limited clay formation to capture Li cations, intermediate *W*/*D* is characterized by high δ^7^Li and reduced Li flux owing to abundant clay formation, and high *W*/*D* is characterized by low δ^7^Li and low Li flux owing to leaching of previously formed clays (*49*, *50*). On short timescales, low *W*/*D* regimes are marked by a coupling between increased river Li flux and decreased δ^7^Li that drives global seawater δ^7^Li toward lower values, whereas high *W*/*D* regimes with low Li flux have less influence on the global marine Li budget and do not generate large negative δ^7^Li excursions (fig. S5). Hence, we attribute the abrupt negative excursion of global seawater δ^7^Li to a rapid decline in *W*/*D* across the P-Tr boundary, indicative of notably decreased weathering intensity and reduced pedogenic clay formation in terrestrial environments. Although the δ^13^C profile of each section is slightly different, close correlations between carbonate δ^7^Li and δ^13^C are observed in the study sections (Fig. 3A), suggesting that decreases in *W*/*D* during the P-Tr transition were tightly related to global climate change.

**Fig. 3.**
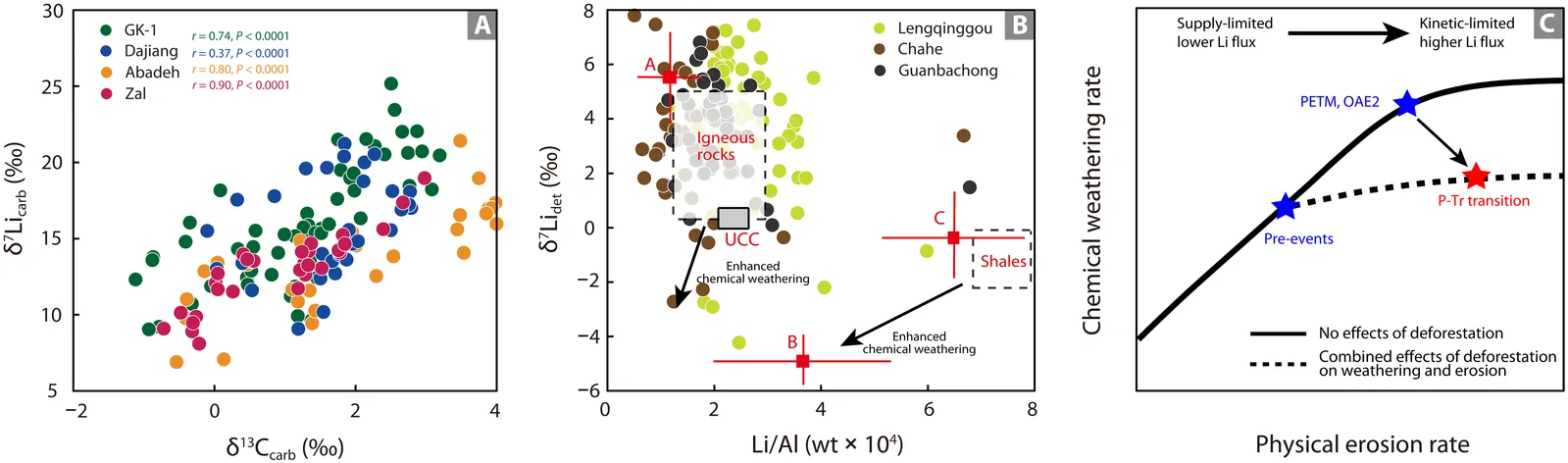
Evaluation of continental silicate weathering evolution during the P-Tr transition. (**A**) Li isotope versus C isotope in marine carbonates. (**B**) Li isotopes versus Li/Al ratios in terrestrial deposits. (**C**) The schematic diagram for sensitivity of weathering to erosion and climate during the P-Tr transition. The areas in (B) represent endmember compositions of igneous rocks (Li/Al = 1 to 3 ppm/wt %, δ^7^Li = +3 to +5‰), average upper continental crust (UCC) (Li/Al = 2 to 3 ppm/wt %, δ^7^Li = 0 to +1.2‰), and shales (Li/Al = 7 to 8 ppm/wt %, δ^7^Li = −2 to 0‰) (*54*). The squares (A, B, and C) in (B) represent endmember compositions of modern riverine detrital sediments under various weathering regimes (*51*). Endmember A represents a physically eroded mafic rock with high δ^7^Li and low Li concentrations (δ^7^Li ≈ +6.2 ‰, [Li] ≈ 11.8 ppm, Li/Al ≈ 2.0 ppm/wt %); endmember B represents a highly congruent weathering product with low δ^7^Li and relatively low Li concentrations (δ^7^Li ≈ −5.0 ‰, [Li] ≈ 37.0 ppm, Li/Al ≈ 3.5 ppm/wt %); and endmember C represents an incongruent weathering product with relatively high δ^7^Li and Li concentrations (δ^7^Li ≈ −1.5‰, [Li] ≈ 62.9 ppm, Li/Al ≈ 7.0 ppm/wt %). In (C), the curves are modified from the weathering model from (*58*), in which the axes are presented as logarithmic scales.

### Detrital δ^7^Li as a record of terrestrial weathering products during the P-Tr transition

To better constrain changes in continental weathering during the P-Tr transition, we generated detrital δ^7^Li records for three terrestrial successions in South China. Compared to the marine carbonate δ^7^Li records, which provide insights into global seawater signals, detrital δ^7^Li records serve as a direct indicator for regional weathering products, reflecting the combined contributions of physical erosion and chemical weathering (*51*, *52*). The terrestrial P-Tr sections in South China represent alluvial and coastal marsh-littoral deposits (*38*), with little influence of marine authigenic clays (*53*). Hence, δ^7^Li signals in these sections denote the relative proportions of unweathered silicate minerals and secondary clay minerals. Compared to continentally formed clays, primary igneous rocks (especially those of mafic composition) have higher δ^7^Li and lower Li contents, providing a promising tool to explore weathering sources and processes on a regional scale (*54*). Despite variability in detrital δ^7^Li signals, terrestrial sections in South China are characterized by consistent increases in δ^7^Li from −2 to 0‰ to +6 to 8‰ following the EPME (Figs. 1C and 2B). Although recent studies have inferred diachronous terrestrial biological and environmental crises during the P-Tr transition, the onset of the terrestrial crisis globally occurred before the P-Tr boundary (*38*, *55*–*57*). While the terrestrial successions likely record a regional environmental response, increased δ^7^Li values in these sections indicate a decline in weathering intensity and more preservation of unweathered silicate minerals following the terrestrial extinction in South China.

Relationships of δ^7^Li to Li/Al in terrestrial detrital materials can be used to evaluate changes in weathering regimes based on modern riverine Li systematics (Fig. 3B). Specifically, igneous rocks generally have high δ^7^Li and low Li/Al, whereas shale has low δ^7^Li and high Li/Al (*54*), allowing assessment of the relative fluxes of Li from these sources (*51*). The detrital data in this study show that, following the EPME on land, silicate weathering regimes changed from high weathering intensity with relatively low δ^7^Li and Li/Al values to lower weathering intensity with higher δ^7^Li and lower Li/Al values. The coupling of high δ^7^Li and low Li/Al observed in the terrestrial sections is similar to the characteristics of modern basaltic terrains subject to low weathering intensity (Fig. 3B), indicating enhanced physical erosion of primary igneous rocks and preservation of unweathered materials in lowermost Triassic terrestrial successions.

### Changes in global continental weatherability linked to end-Permian deforestation

The δ^7^Li data from both marine and terrestrial records in this study consistently indicate decreased weathering intensity and reduced clay formation at both global and regional scales, coincident with the marine and terrestrial extinctions at the P-Tr boundary (Fig. 2). Other hyperthermal events during the Phanerozoic (e.g., Cretaceous OAE2 and PETM) (*28*, *41*) were also marked by large negative δ^7^Li excursions of global seawater, which have been interpreted as a result of decreased weathering intensity. Reduced weathering intensity during the PETM and OAE2 events has been attributed to a greater increase in physical erosion relative to chemical weathering due to intensification of the hydrological cycle and hyperwarming (Fig. 3C) (*58*). This scenario implies that an elevated chemical weathering rate was related to climatic warming, which in turn facilitated the stabilization of extreme climate conditions via consumption of atmospheric CO_2_. Compared with these hyperthermal events, the P-Tr transition was marked by prolonged elevation of atmospheric CO_2_ levels and extreme temperatures following the initial phase of rapid volcanic degassing in the latest Permian (*12*, *13*, *19*).

The persistence of high atmospheric CO_2_ through the Early Triassic was likely linked to a muted feedback of silicate chemical weathering rates on Early Triassic global warming, which we infer was owing to suppressed chemical weathering and atmospheric CO_2_ consumption. Here, we propose that widespread terrestrial deforestation during the P-Tr transition likely plays a critical role in shaping chemical weathering rates (Fig. 3C). Despite the potential diachroneity of the terrestrial biological and environmental crises (*38*), deforestation occurred globally during the P-Tr transition, likely due to extreme climate perturbations, ozone collapse–driven ultraviolet irradiation, acid rain, and widespread wildfires (*16*, *59*). The onset of terrestrial crisis and deforestation at a global scale is considered to have occurred prior to the marine extinction, especially at middle to high paleolatitudes (*55*, *57*, *60*). Following decimation of gymnosperm forests during the EPME, terrestrial floras underwent multiple transitions during the Early Triassic. The end-Permian biocrisis coincided with a fungal spike and abundant organic debris with evidence of greatly increased wildfire activity (*61*, *62*). The earliest Triassic witnessed the spread of a disaster flora dominated by the seed fern *Peltaspermum* at both low and high paleolatitudes (*59*, *63*). Over the next several hundred thousand years, a successional recovery of terrestrial floras took place, marked by pioneering herbaceous lycopsids such as *Annalepis* and *Pleuromeia*, followed by conifer forests in the early Middle Triassic (*63*, *64*). Overall, the resurgence of forests and terrestrial ecosystems was notably delayed after the EPME, and the low–carbon-density character of the Early Triassic floras resulted in a “coal gap” linked to the absence of peat-forming plants (*65*), a feature that limited their ability to mediate weathering reactions and consumption of atmospheric CO_2_.

Colonization of a land surface by plants, particularly vascular plants, has been shown to accelerate the chemical weathering of minerals and rocks (i.e., increased weatherability) and detrital clay formation based on both laboratory experiments (*66*, *67*) and natural observations (*68*–*70*), and this relationship has been widely used in chemical weathering models (*71*, *72*). Despite some uncertainties regarding the latitudinal influence of land plants on regional physical erosion rates, forested areas on a global scale are generally characterized by lower erosion rates than barren surfaces, primarily due to reduced soil erodibility (*73*, *74*). Both ancient and modern investigations have demonstrated that afforestation can substantially influence continental chemical weathering process and Li isotope fractionation at a range of timescales (*75*, *76*). In contrast, deforestation and collapse of terrestrial ecosystems during the EPME have theoretically reduced chemical weathering rates and enhanced physical erosion rates, resulting in rapidly decreased weathering intensity during the P-Tr transition. Additionally, due to changes in the hydrological cycle and increased surface temperature, shifts in fluvial styles and increased terrestrial sediment fluxes to the ocean are thought to have occurred during the P-Tr transition (*4*, *16*), consistent with a transition to a kinetically limited weathering regime and decreased global continental weatherability in the aftermath of the end-Permian crisis. The effects of global deforestation on continental weathering are further tested in an Earth system model below.

### Modeling silicate weathering changes during the P-Tr transition

We used the Spatial Continuous Integration (SCION) biogeochemical model, which combines spatial representations of climate and continental processes based on the GEOCLIM model (*77*) and long-term biogeochemical cycles based on the COPSE model (*78*), to evaluate changes in continental silicate weathering and its effects on global seawater chemistry. Compared to previous models that calculate silicate weathering rates as functions of atmospheric CO_2_ level (e.g., LOSCAR) or a combination of temperature and runoff (e.g., cGENIE), the SCION model allows for more detailed approximations of continental weathering processes through variations of temperature, physical erosion, and runoff (as in GEOCLIM) as well as biotic effects (as in COPSE). In this study, we used the SCION model to simulate the global climatic and environmental changes during the P-Tr transition, incorporating additional carbon injection and reduced influence of land plants on global silicate weathering. The duration and amount of CO_2_ emissions in the latest Permian were independently modified from previous estimates (Fig. 4A) (*18*, *79*). Changes in global land plant productivity and its effect on chemical weathering were modified from a recent SCION model investigation that independently evaluated the land plant distributions from the latest Permian to the Middle Triassic (Fig. 4B) (*80*). Temporal changes in weathering intensity during the P-Tr transition are calculated by global average ratios of silicate chemical weathering and physical erosion rates derived from the SCION model.

**Fig. 4.**
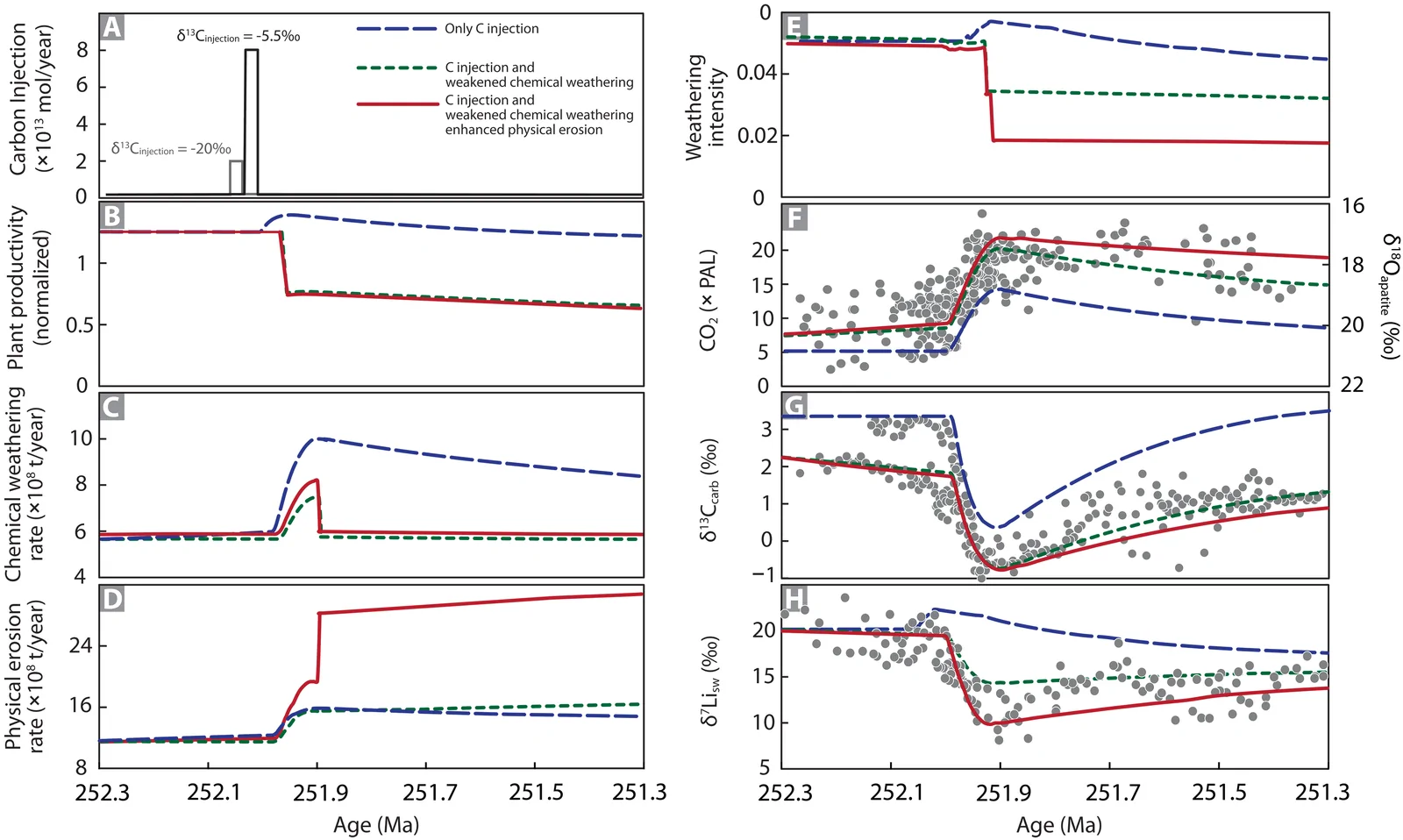
Changes in key environmental factors to the SCION model and patterns of silicate weathering and marine chemistry evolution during the P-Tr transition. (**A** and **B**) Parameter changes for carbon injection to the atmosphere (A) and changes in land plant productivity relative to the modern level (B). (**C** to **H**) Results for chemical weathering rate (C), physical erosion rate (D), weathering intensity (E), atmospheric CO_2_ level (F), marine carbonate δ^13^C records (G), and seawater δ^7^Li records (H) under different conditions, including only increased carbon injection, increased carbon injection and weakened chemical weathering due to deforestation, and increased carbon injection and coupling of weakened chemical weathering and enhanced physical erosion due to deforestation. Isotopic data in (G) and (H) are from this study. Global conodont apatite O isotope data in (F) are compiled from (*12*).

Our model yielded chemical weathering and physical erosion rates, weathering intensity, atmospheric CO_2_ level, seawater inorganic δ^13^C, and seawater δ^7^Li records (Fig. 4, C to H). Global seawater δ^7^Li was calculated using a Li mass balance model, based on modern relationships of riverine δ^7^Li to weathering intensity (*49*) and Li isotope fractionations between its sink and seawater (see the Supplementary Materials for detailed methods). Three types of climatic and environmental conditions were simulated, including (i) increased carbon injection only, (ii) increased carbon injection and weakened chemical weathering due to the deforestation, and (iii) increased carbon injection and coupling of weakened chemical weathering and enhanced physical erosion due to deforestation. The consideration of CO_2_ emissions alone can lead to rapid increases in atmospheric CO_2_ (Fig. 4F), along with negative marine δ^13^C_carb_ excursions during the P-Tr transition (Fig. 4G), consistent with patterns for several hyperthermal events during the Phanerozoic (*81*). However, in the present simulation, perturbations of global climate and environment were efficiently stabilized via an enhanced silicate weathering feedback (Fig. 4), which is opposed to geochemical records documenting persistently high atmospheric CO_2_, global warming, and marine anoxia in the earliest Triassic (*7*, *13*, *14*). Additionally, the SCION model also showed limited responses of *W*/*D* in this scenario, likely reflecting the balance between chemical weathering and physical erosion due to increased temperature and runoff. In contrast, with the addition of the terrestrial vegetation collapse and associated responses of chemical weathering and physical erosion, the model results show notably decreased *W*/*D* and delayed decreases in atmospheric CO_2_, corresponding to widespread deforestation after CO_2_ emission during the P-Tr transition. The global marine δ^13^C and δ^7^Li outputs are consistent with carbonate records that are marked by substantially lower baseline values of δ^13^C and δ^7^Li in the earliest Triassic than those in the latest Permian, consistent with a delayed recovery of climatic and environmental conditions globally during the earliest Triassic (Fig. 4, F to H). In particular, abrupt negative δ^7^Li excursions of ∼10‰ during the P-Tr transition and persistent low δ^7^Li signals during the earliest Triassic require coupled responses of chemical weathering and physical erosion to terrestrial deforestation (Fig. 4H). Despite extreme ecological and environmental fluctuations through the Early Triassic, our hypothesis of decreased weathering intensity and suppressed chemical weathering rate accounts for observed changes in global climate and environment well, and our model faithfully reproduces changes in oceanic isotopic records during the P-Tr transition.

Evidence for reduced weathering intensity and a suppressed chemical weathering response to global warming during the P-Tr transition is consistent with global seawater ^87^Sr/^86^Sr evolution and regional sedimentary CIA records. Compared to δ^7^Li records, seawater ^87^Sr/^86^Sr shows only limited variation through the P-Tr transition, followed by a pronounced increase from the late Induan to Olenekian, ∼2 Myr after the P-Tr boundary (Fig. 2E and fig. S6) (*26*, *27*, *82*, *83*). This delayed rise indicates that enhanced chemical weathering did not immediately respond to global warming, consistent with a transient reduction in continental weatherability, likely associated with widespread terrestrial deforestation. In addition to chemical weathering rate, the change in weathered lithology (e.g., more mafic weathering) and shift of weathering zones poleward due to the deforestation have also been considered to influence seawater ^87^Sr/^86^Sr during the Early Triassic, complicating the use of ^87^Sr/^86^Sr as a direct proxy for chemical weathering rate (*80*). More high-resolution records of paired ^87^Sr/^86^Sr and δ^7^Li data may provide further constraints on global continental weathering through the Early Triassic. Meanwhile, sedimentary CIA and clay mineral records show no substantial increase in weathering intensity across the P-Tr boundary (*25*), reinforcing the interpretation of a globally suppressed chemical weathering response during the initial phase of the P-Tr transition.

### Sustained Early Triassic crisis linked to depressed global continental weatherability

Compared to other hyperthermal events during the Phanerozoic that were associated with enhanced volcanic activity, the P-Tr transition experienced a more prolonged interval of environmental and biological crisis, which profoundly influenced Earth’s habitability (*81*). In other volcanic-induced hyperthermal events (e.g., Cretaceous OAE2 and PETM), Earth’s weathering regulation system was not as drastically impaired, allowing for a quicker stabilization of climate once volcanic activity subsided (*19*). However, rapid volcanic carbon emissions during the P-Tr transition cannot solely explain prolonged global warming and high atmospheric CO_2_ levels through the Early Triassic (Fig. 4). Here, we use Li isotope records and a modified biogeochemical model to verify the hypothesis that reduced sequestration of atmospheric CO_2_ through silicate weathering and terrestrial organic carbon burial occurred as a consequence of widespread deforestation and decreased weatherability in the earliest Triassic. Although the span of Li isotope data and modeling results in this study only directly identified the effects of terrestrial deforestation on continental weathering and carbon cycle during the P-Tr transition, this effect may have been sustained through the Early Triassic until the recovery of terrestrial forests (*63*, *64*, *80*) and accounted for the delayed shift in seawater ^87^Sr/^86^Sr values, which amplified the global climatic and environmental perturbations of the Early Triassic. In this view, the depressed response of continental weathering to global warming may have facilitated prolonged high atmospheric CO_2_ and greenhouse conditions during the Early Triassic.

Despite the fact that decreases in weathering intensity and chemical weathering rate likely result in declines in phosphorus weathering in silicate rocks (*84*), deforestation and reduced clay formation may have inhibited the recycling and uptake of phosphorus in soils and led to a transient spike of particulate and organic phosphorus into river systems, ultimately accelerating phosphorus transport to the oceans (*85*–*87*). Moreover, decreased terrestrial clay formation and transport to continental shelf oceans reduced the burial efficiency of marine organic matter, promoting the remineralization of organic carbon and consumption of dissolved O_2_ in the oceanic water column (*52*, *88*). All of these processes exacerbated global marine anoxia beyond the direct effects of high temperature conditions on oceanic deoxygenation, likely different from other hyperthermal events during the Phanerozoic (e.g., PETM) (*89*). Together, depressed continental weatherability associated with widespread deforestation may have been a key cause of persistently high atmospheric CO_2_ and global temperatures during the P-Tr transition, which also led to intensified Earth system instability and delayed biotic recovery during the Early Triassic (*3*, *90*). This study underscores the critical role of terrestrial vegetation in shaping Earth’s long-term carbon cycle and highlights the value of integrating isotope proxies and numerical modeling to reconstruct the evolution of climate and marine environment in deep time.

## METHODS

### Geochemical analysis

Four marine carbonate sections in South China, central and northwestern Iran, and western Austria, and three terrestrial sections in South China were investigated for Li isotopes in this study (see the Supplementary Materials for sample details and stratigraphic correlations). Sample preparation and elemental and Li isotopic measurements were performed at the Center for Research and Education on Biological Evolution and Environment (CREBEE), Nanjing University, following the methods in (*33*). The Li isotopes were measured on a Thermo Fisher Scientific Finnigan Neptune XT MC-ICP-MS at CREBEE, Nanjing University. An in-house Li solution (HPS-Li_NJU_, δ^7^Li = +20.4 ± 0.4‰, 2σ, *n* = 30) was used to monitor the long-term reproducibility of Li isotope analyses on the machine. The overall reproducibility and accuracy of the total procedure (sample digestion, Li separation, and isotope measurement) were checked by repeated analyses of GSR-12 (dolomite, [Li]_leachate_ = 1.6 μg/g, δ^7^Li = +13.6 ± 0.5‰, 2σ, *n* = 5) and OSIL seawater (δ^7^Li = +30.8 ± 0.4‰, 2σ, *n* = 5), with different aliquots processed through column chemistry.

### Modeling simulations

The numerical model SCION is a climate-enabled biogeochemical model that runs over Phanerozoic timescales, which works as a “bridge” between global biogeochemical box models and spatially resolved Earth system models. In this study, the recent baseline version of SCION (https://github.com/bjwmills/SCION) (*78*) and a modified version of SCION for the P-Tr terrestrial plant crisis (https://github.com/ZhenXuJane/SCION_Xu2025) (*80*) were used to evaluate changes in global physical erosion and chemical weathering rates, as well as C-Li isotope cycling during the P-Tr transition. For further information, see the detailed model descriptions in the Supplementary Materials.

## Supplementary Materials

### This PDF file includes:

Supplementary Text Figs. S1 to S7 Tables S1 Legend for Table S2 References
