# Supplementary material for: Depressed chemical weathering inhibited climate stabilization after the end-Permian biocrisis
Source: Sci Adv. 2026 Oct 28;12(44):eaee6350. doi: 10.1126/sciadv.aee6350 (PMC13718703; doi:10.1126/sciadv.aee6350)
Supplement: Supplementary file 1 [file sciadv.aee6350_sm.pdf]

Science Advances
AAAS

Supplementary Materials for

Depressed chemical weathering inhibited climate stabilization after the end-Permian biocrisis

Guang-Yi Wei *et al.*

Corresponding author: Feifei Zhang, fzhang@nju.edu.cn

Sci. Adv. **12**, eaee6350 (2026)
DOI: 10.1126/sciadv.aee6350

This PDF file includes:

Supplementary Text
Figs. S1 to S7
Tables S1
Legend for table S2
References

S1. Supplementary Text

1.1 Geological settings and age correlations

In this study, marine carbonate samples were collected from three widely separated regions around the Tethys Ocean—Dajiang section from the South China, Abadeh and Zal sections from the central and northwestern Iran, and GK-1 drillcore from the western Austria. In South China, the Dajiang section was exposed in an exceptionally preserved carbonate platform—Great Bank of Guizhou, Nanpanjiang Basin, South China Block, which is lied in equatorial latitudes and isolated from the Pangea by the Paleotethys Ocean to the west and the Panthalassa Ocean to the east (*3*). The Dajiang section was located at the platform interior, recording the full interval spanning from the latest Permian to the early Triassic. The uppermost Permian fossiliferous limestone is overlain by ~15 m microbialite at the Permian−Triassic boundary. Lower Triassic strata continue with ∼50 m of thin-bedded limestones. We correlate the Dajiang section to the GSSP Meishan section, using biostratigraphy and isotope chemostratigraphy and age assignment was adopted by documented ages from Meishan. These two sections have been dated via conodont biostratigraphy and carbon isotopic chemostratigraphy, and tentatively correlated with shallow-marine Tethyan carbonate sections, which employ conodont biostratigraphy (*92*). In Iran, the investigated Abadeh section is located at the Hambast Valley, about 35 km SE of Abarkuh town and about 60 km southeast of Abadeh town in the central Iran, and the Zal section is located at the Zal Mountain, about 2.4 km of Zal village in the northwestern Iran, which are located at a pelagic setting as an isolated block in the western part of Tethys Ocean (*39, 93, 94*). In the Abadeh and Zal sections, the upper Permian strata are composed of thin-bedded gray or reddish limestone with abundant ammonoids and conodonts, and the lower Triassic strata are composed of light gray limestones of the *Claraia* Beds with the basal Triassic conodont zone at its base. The Permian-Triassic boundary in the Abadeh and Zal sections is marked by a very thin "Boundary Clay" (~0.5 m thick), consisting of dark red and green shales with few intercalations of very thin greenish or gray wackestone (*94*). In western Austria, the GK-1 drillcore is located at the Southern Alps, deposited on a carbonate

ramp near the western edge of the paleo-Tethys Ocean (*95, 96*). The uppermost Permian to the lower Triassic strata of the GK-1 drillcore consist of micritic or microsparitic light gray dolostones, representing the syn-sedimentary dolostone deposits in shallow-marine subtidal to supratidal environment (*96*). The Permian-Triassic sequences in shallow-marine carbonate sections have been the subject of extensive research for several decades, facilitating the precise determination of biostratigraphical, chemostratigraphical and chronostratigraphic ages through the utilization of detailed markers, including zircon U-Pb dating, conodont zonation and C isotopes (Fig. S1) (*39, 93, 94*).

The terrestrial sections in this study are located on the eastern flank of the Emeishan Large Igneous Province on the western margin of the South China Block, including Guanbachong, Chahe, and Lengqinggou sections from onshore to paralic settings (*38*). In this region, the uppermost Permian to the lowest Triassic strata are composed of the Xuanwei Formation, Kayituo, and Dongchuan or Feixianguan formations in ascending order. The Xuanwei Formation consists of alluvial deposits interlayered with organic-rich mudstones or coal seams. The overlying Kayitou Formation mainly consists of alternative olive or maroon siltstones or sandstones with relatively abundant plant fossils in the lower part, and breccia and pedogenic calcareous nodules in the upper part. The Dongchuan Formation in the Chahe and Guanbachong sections consist of purple-red sandstones and mudstones with numerous pedogenic nodules and breccia. Comparably, the Feixianguan Formation in the Lengqinggou section consists of calcareous mudstones and limestones, representing a coastal transitional environment. The accurate depositional age and P-Tr boundary in the terrestrial sections from the South China are modified from Zircon U-Pb dating in these sections (*38, 40*). The correlations among three terrestrial sections are mainly derived from the organic carbon isotope records that consistently exhibit negative excursions at the upper part of the Kayitou Formation (*40*). The extinction interval in these terrestrial sections is identified based on land plant fossil ranges in each section, beginning at the lower part of the Kayitou Formation below the onset of negative excursions of organic carbon isotopes (*40*).

1.2 Geochemical analysis of marine carbonates and terrestrial deposits

For carbonate samples, approximately 60-100 μg of powder was used to analyze C and O isotopes by a Thermo Scientific Delta V mass spectrometer at the State Key Laboratory of Critical Earth Material Cycling and Mineral Deposits, Nanjing University. Isotope values are presented as per mil variations (‰) relative to the Vienna Pee Dee belemnite (VPDB) standard. The external analytical precision for C and O isotopes is monitored based on the repeated analyses of standard GBW04417 ($\delta^{13}C_{carb}$ = -6.06 ± 0.13‰, $\delta^{18}O_{carb}$ = -24.12 ± 0.5 ‰ 2SD). The element and Li isotopic analyses were treated with a well-developed sequential extraction procedure, in order to eliminate exchangeable cations and accurately extract the elements (e.g., Li) fraction in lattices of carbonate minerals. These chemical treatments were conducted at the Metal Isotope Geochemistry Lab in Centre for Research and Education on Biological Evolution and Environment (CREBEE), Nanjing University. The procedure is described briefly: approximately 200 mg powder was weighed in centrifuge tubes, 10 mL Milli-Q water was added in to dissolve water-soluble salts for 2 hours on shaker in 75 rpm at room temperature, then the supernatant was separated by centrifugation and repeated two times. Then the residue was further extracted with 15 mL of 1.0 M ammonium acetate buffer solution (pH of this buffer solution was adjusted to ~7.5-8.0, in order to not leach Li in carbonate lattice) with continuous shaking for 4 h at room temperature, to target exchangeable cations and then the supernatant was separated as described above. The residue after extracting for exchange fraction was conducted with 25 mL of 0.75 M acetic acid solution (~5% by volume). The mixture was continuously ultrasound shaken for 1h in room temperature and nearly all carbonate minerals were dissolved after another 4 hours at constant agitation speed. Lithium extracted in this fraction is mainly associated with carbonate minerals. The resulting solution was separated in Teflon beaker and dried on heater at 100°C, then the final mixture materials were re-dissolved and stored in 6 M HCl as a stock solution for elemental concentration and isotope determination. For siliciclastic samples, approximately 50 mg powder was proactively leached with 2 M HCl for 0.5 h to remove potential carbonate minerals in the samples, then fully digested in Teflon beakers using distilled mixing acids of HF-HNO_3-HCl. The sample solution was re-dried on heater at 100°C, then the final mixture materials were re-dissolved and stored in 6 M HCl as a

stock solution for elemental concentration and isotope determination.

Major and trace elemental concentrations were measured using a Thermo Fisher iCap Pro OES and Element XR at the Metal Isotope Geochemistry Lab, CREBEE, Nanjing university. The typical precision was better than 5 % for major elements and 10 % for trace elements, based on repeated analysis of in-run check standards. For Li isotopic analysis, a single-column method with AG50W-X12 cation resin and 0.2 N HCl was used to purify Li from the solution matrix(*33*). The purified sample solution was then measured on a Thermo Scientific Finnigan Neptune XT MC-ICP-MS at the CREBEE, Nanjing University. An in-house Li solution (HPS-Li, δ^7Li = 20.4‰ ± 0.4‰, 2σ, *n* = 30) was used to monitor the long-term reproducibility of Li isotope analyses on the machine. The overall reproducibility and accuracy of the total procedure (sample digestion, Li separation and isotope measurement) were checked by repeated analyses of GSR-12 (dolomite, $[Li]_{leachate}$ = 1.6 μg/g, δ^7Li = 13.6 ± 0.5‰, 2σ, n=5) and OSIL seawater (δ^7Li = 30.8 ± 0.4‰, 2σ, *n* = 5), with different aliquots processed through column chemistry.

1.3 Effects of detrital contamination, carbonate mineralogy and early diagenesis on carbonate δ^7Li records

Lithium contamination from detrital materials during bulk carbonate digestion can significantly bias carbonate δ^7Li signatures, as detrital aluminosilicates (e.g., clays) contain substantially higher Li concentrations than carbonate minerals. To assess potential detrital contributions, the molar ratios of Al/(Ca+Mg) in carbonate leachates following sequential dissolution have been widely used as indicators of aluminosilicate contamination. In our sequential leaching experiments using weak acetic acid, most carbonate fractions exhibit low Al/(Ca+Mg) ratios (Fig. S2A), well below the recommended threshold of 0.8 mmol/mol established in previous studies of ancient carbonates (*28, 97*). The study samples also display low Mn/(Ca+Mg) ratios (Fig. S2B), suggesting minimal dissolution of Mn oxides and limited Li addition from such phases. Moreover, the absence of significant correlations between δ^7Li and either Al/(Ca+Mg) or Mn/(Ca+Mg) further indicates that detrital contamination does not

appreciably affect the δ^7Li signatures of the analyzed carbonates.

The Li isotope fractionations between carbonate and seawater should also be fully constrained when using marine carbonate to reconstruct δ^7Li of coeval seawater. The carbonate mineralogy and early diagenetic process have been considered to display a first-order control on Li isotopic fractionations between natural carbonate and seawater (*29, 33, 98*). Primary aragonite typically exhibits substantially larger Li isotope fractionations relative to primary calcite (*30, 99*), whereas early diagenetic alteration generally reduces these differences and drives the δ^7Li values of carbonate rocks towards those of coeval seawater (*32, 33*). The study sections comprise three limestone successions in outcrop (Dajiang, Abadeh, and Zal) and one dolostone drillcore (GK-1). All carbonates from these sections display markedly low Sr/(Ca+Mg) ratios (~0.01–1 mmol/mol; Fig. S4D), indicating the absence of preserved primary aragonitic signals and seawater-buffered conditions during early diagenesis (*33*). Consistently low Li/(Ca+Mg) ratios (<10 μmol/mol) further support strongly seawater-buffered conditions (*33*). In this view, the carbonates are more likely to record δ7Li values representative of coeval seawater with limited isotopic fractionation; here, we adopt a reference value of –2‰ based on late Cenozoic shallow-marine carbonate studies (*29, 32, 33*). Importantly, despite differences in lithology (limestone versus dolostone), all four sections exhibit highly consistent δ^7Li variations, with no significant correlations between δ^7Li and Mg/Ca, Li/(Ca+Mg), Sr/(Ca+Mg), or $\delta^{18}O$ values (Fig. S4). Collectively, these observations suggest that the observed δ^7Li variations primarily reflect changes in global seawater composition across the Permian–Triassic transition, rather than local depositional or diagenetic effects.

1.4 Constraining the evolution of continental silicate weathering using a SCION Model

The standard runs of SCION were performed using the current basline version of the model (v1.2.0, code in MATLAB, https://github.com/bjwmills/SCION) (*78*). The SCION model works as an amalgamation of the spatially resolved GEOCLIM model for hydrological and climate conditions (*100*), and the COPSE model for global biogeochemical cycles (*101*). The

baseline SCION results from ca. 600 Ma to present day are shown in previous work (*78*), and then cut between 252. 3 Ma to 251.3 Ma for the 'C injection only' scenario for the Permian-Triassic transition in this work. The nondimensional forcing factors, including degassing, land area of different lithologies, plant evolution, and plant effects on weathering are derived from "COPSE reloaded" model (*101*). Based on basic frameworks from COPSE reloaded model, the evolution of land plants (E) initially increases from 0 to 0.15 over the interval from 465 Ma to 445 Ma, and then from 0.15 to 1 over the interval from 400 Ma to 350 Ma that is associated with deep-rooted vascular plants. The plant effect on weathering (W) initially increases from 0 to 0.75 over the interval from 465 Ma to 445 Ma, and then from 0.75 to 1 over the interval from 400 Ma to 350 Ma that is associated with deep-rooted vascular plants. For the environmental perturbations across the Permian-Triassic boundary, we added extra carbon injection beyond the baseline degassing for short timescales, following refs. (*18, 63*). Two phases of rapid carbon injection with distinct $\delta^{13}C$ values were simulated in the model—2×10^{13} mol/yr with a $\delta^{13}C$ value of –20‰ for 50 kyr, and 8×10^{13} mol/yr with a $\delta^{13}C$ value of –5.5‰ for 40 kyr (Fig. 4A). To consider the terrestrial extinction and forest ecosystem collapse, we run the modified version of SCION with an independent spatial reconstruction of global land plant distribution and productivity from the Late Permian to the Early Triassic (code in MATLAB, https://github.com/ZhenXuJane/SCION_Xu2025) (*80*). The effects of land plants, especially forests, on continental silicate weathering (f_{biota}) are estimated to be associated with regional Net Primary Productivity of land plants after the CO_2 fertilization effect ($NPPL_f$) based on the equation in ref. (*80*).

$$f_{biota} = 0.002 \cdot NPPL_f + 0.25$$

Here, the colonization of deep-rooted plants is considered to enhance chemical weathering (*67, 68, 72*) and also to inhibit physical erosion (*102-104*) under the greenhouse climate conditions,

$$W = f_{biota} \cdot W0, \quad and \quad E = E0 \, / \, f_{biota}$$

where W0 and E0 denote present day global weathering and physical erosion rates. The choice of 0.25 for f_{biota} represents the baseline of weathering and erosion without land plants in COPSE-reloaded and CEOCARB III models (*101, 105*). With the scaling factor of 0.002, the modeling results with present day integration of $NPPL_f$ reproduce present day global weathering and erosion rates (i.e., $f_{biota} = 1$) (*80*).

After adding the new forcing factors in SCION, we re-ran the model in MATLAB using the variable-step variable-order implicit routine for stiff systems (ODE15s) with a time interval maximum of 10^5 yr, and generated the results of atmospheric CO_2, $\delta^{13}C_{carb}$, silicate weathering flux, and physical erosion flux.

1.5 Modeling riverine and marine δ^7Li curves, based on W/D results from SCION model

The riverine δ^7Li (δ^7Li_{riv}) and Li flux from silicate weathering (F_{riv}) was calculated as a function of W/D in the weathering zone, following refs. (*106, 107*).

$$\delta^7 Li_{riv} = -\frac{E}{W}\left(\delta^7 Li_{sed} - \delta^7 Li_{rock}\right) + \delta^7 Li_{rock} \quad (1)$$

$$\frac{E}{W} = \frac{(1-W/D)}{W/D} \quad (2)$$

Here, W/D for the Li system is assumed to be equivalent to *W/D* for overall silicate weathering(*107*). Although this assumption is a simplification, it can provide a reasonable fit for the typical 'boomerang' pattern observed in modern δ^7Li_{riv} databases (*107*) (also see our new parameterized calculation with published δ^7Li_{riv} data in Fig. S5). The δ^7Li_{rock} represents the Li isotope compositions of bedrocks.

Sedimentary δ^7Li (δ^7Li_{sed}) can be also calculated as a function of *W/D* with a negative relationship, based on data from modern riverine detrital sediments (*108*), parameterized as:

$$\delta^7 Li_{sed} = a \cdot e^{-\frac{b}{W/D}} + \delta^7 Li_{rock} \quad (3)$$

where *a* and *b* are nondimensional constants (see Supplementary Table S1 for detailed parameters) which are calculated by the fit to data from modern riverine detrital sediments (*108*).

Combing equations (1), (2), (3), we can obtain:

$$\delta^7 Li_{riv} = -\frac{(1-W/D)}{W/D}\left(a \cdot e^{-\frac{b}{W/D}}\right) + \delta^7 Li_{rock} \quad (4)$$

Equation (4) can reproduce the overall trend of modern $\delta^7 Li_{riv}$ as a function of *W/D*, with the highest $\delta^7 Li_{riv}$ value corresponding to medium *W/D* (~0.05) (Fig. S5).

To calculate global average *W/D* in deep time based on results from the SCION model, the first step is to estimate the Total Dissolved Solid (*TDS*) flux (in 10^6 t/yr) from silicate weathering using $TDS = (550/8700) \times silw$. The TDS is calculated from the concentrations of major dissolved elements (SiO_2, Ca, Mg, Na, K, SO_4) from silicate weathering. The *silw* (in 10^9 mol/yr) is the CO_2 consumption flux via silicate weathering computed by SCION, and *550/8700* is the ratio of modern world-average estimates of *TDS* flux (in 10^6 t/yr) and CO_2 consumption flux (in 10^9 mol/yr) based on the chemistry of large rivers(*109*). The weathering intensity (*W/D*) is the calculated as *TDS/(TDS+E)*, with the physical erosion flux (*E*) computed by SCION model in t/yr.

The global average Li flux (*Friv*) is also related to *W/D* as an overall negative relationship (Fig. S5B), which can be calculated as functions of $\delta^7 Li_{riv}$.

$$F_{riv} = TDS \cdot \left(\frac{[Li]}{[TDS]}\right) \cdot e^{\frac{\delta^7 Li_{riv} - \delta^7 Li_{rock}}{\Delta_{sec}}}$$

The parameters of this model are *a* (= 4.5‰), *b* (= 0.06), the Li isotope fractionation during incorporation in secondary phases Δ_{sec} (= -17‰), the Li concentration in bedrock *[Li]* (= 30.5 μg/g), and the Li isotopic ratio in bedrock $\delta^7 Li_{rock}$ (= 1.0‰) (*107*). The concentration of major dissolved elements (SiO_2, Ca, Mg, Na, K, SO_4) in bedrock *[TDS]* (= 74.3%), derived from compositions of the average upper continental crust (*110*), is used to scale the Li flux to the silicate weathering flux.

After obtaining W/D results from the SCION model run, we can calculate both $\delta^7 Li_{riv}$ and Li flux. Then, we further calculate global seawater $\delta^7 Li$ changes based on a one-box marine Li isotope mass balance model, presented as:

$$\frac{dN_{Li}}{dt} = F_{riv} + F_{hyd} - F_{AOC} - F_{MAAC} \tag{5}$$

$$N_{Li} \cdot \frac{d\delta^7 Li_{sw}}{dt} = (\delta^7 Li_{riv} - \delta^7 Li_{sw}) \cdot F_{riv} + \left(\delta^7 Li_{hyd} - \delta^7 Li_{sw}\right) \cdot F_{hyd} - (\delta^7 Li_{AOC} - \delta^7 Li_{sw}) \cdot F_{AOC} - (\delta^7 Li_{MAAC} - \delta^7 Li_{sw}) \cdot F_{MAAC} \tag{6}$$

where each $\delta^7Li_{sink} - \delta^7Li_{sw}$ is equal to Δ^7Li_{sink}, N_{Li} is the global marine Li reservoir, F is the flux of marine Li sources and sinks, including river (F_{riv}), high-temperature hydrothermal fluid (F_{hyd}), altered oceanic crust (F_{AOC}), and marine authigenic aluminosilicate clay (F_{MAAC}). The Li sink fluxes are assumed to have a first-order dependence on the marine Li concentration with a partition constant K.

$$F_{AOC} = K_{AOC} \cdot N_{Li}\, ;\ F_{MAAC} = K_{MAAC} \cdot N_{Li} \tag{7}$$

The fluxes and isotopic compositions of MAAC and AOC are assumed to be comparable to Pre-Cenozoic background conditions (*76, 97*), while each Δ^7Li_{sink} is assumed to decrease by ~2‰ at the Permian-Triassic boundary, representing response to increased seawater temperature.

All the parameters used in the model are presented in Supplementary Table S1.

S2. Supplementary Figures

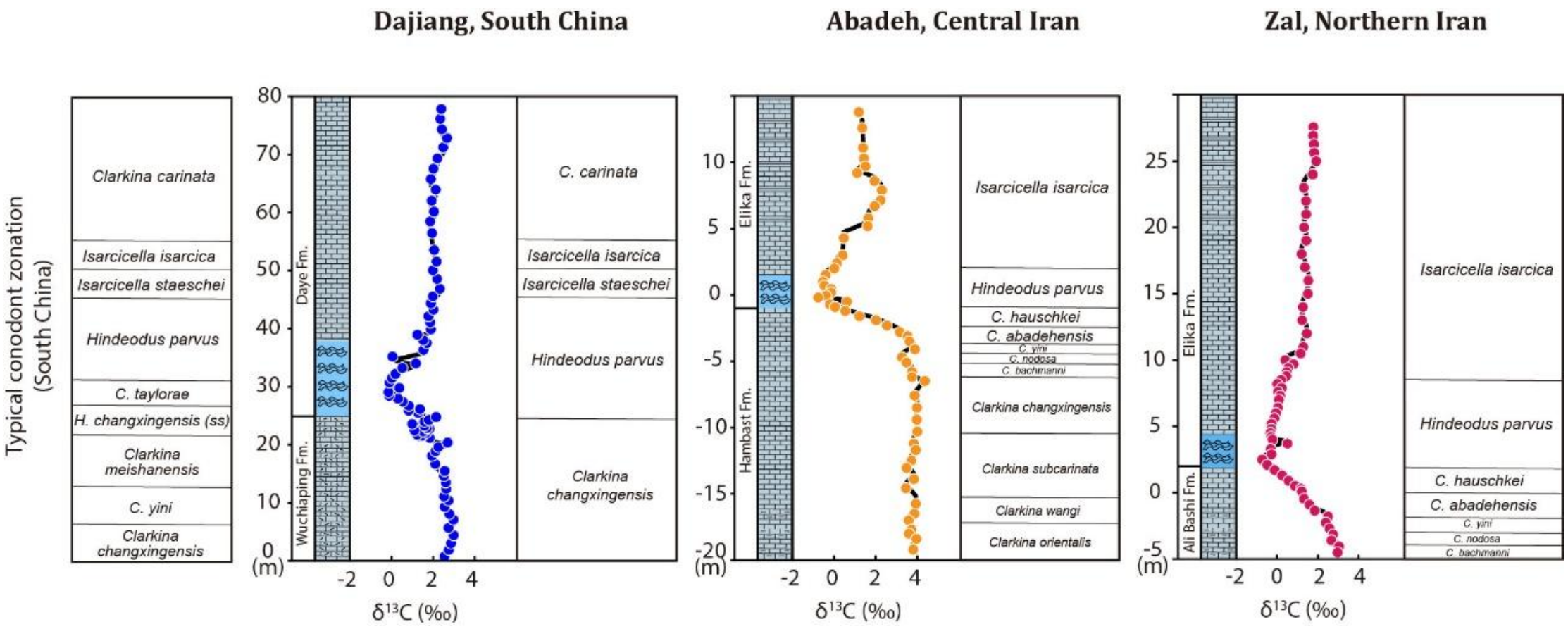

Figure S1. Brief stratigraphic correlations of the study carbonate sections. Records of inorganic carbon isotope and conodont zonation at Dajiang, South China, Abadeh and Zal sections, Iran for stratigraphic correlations (*35, 39, 111*).

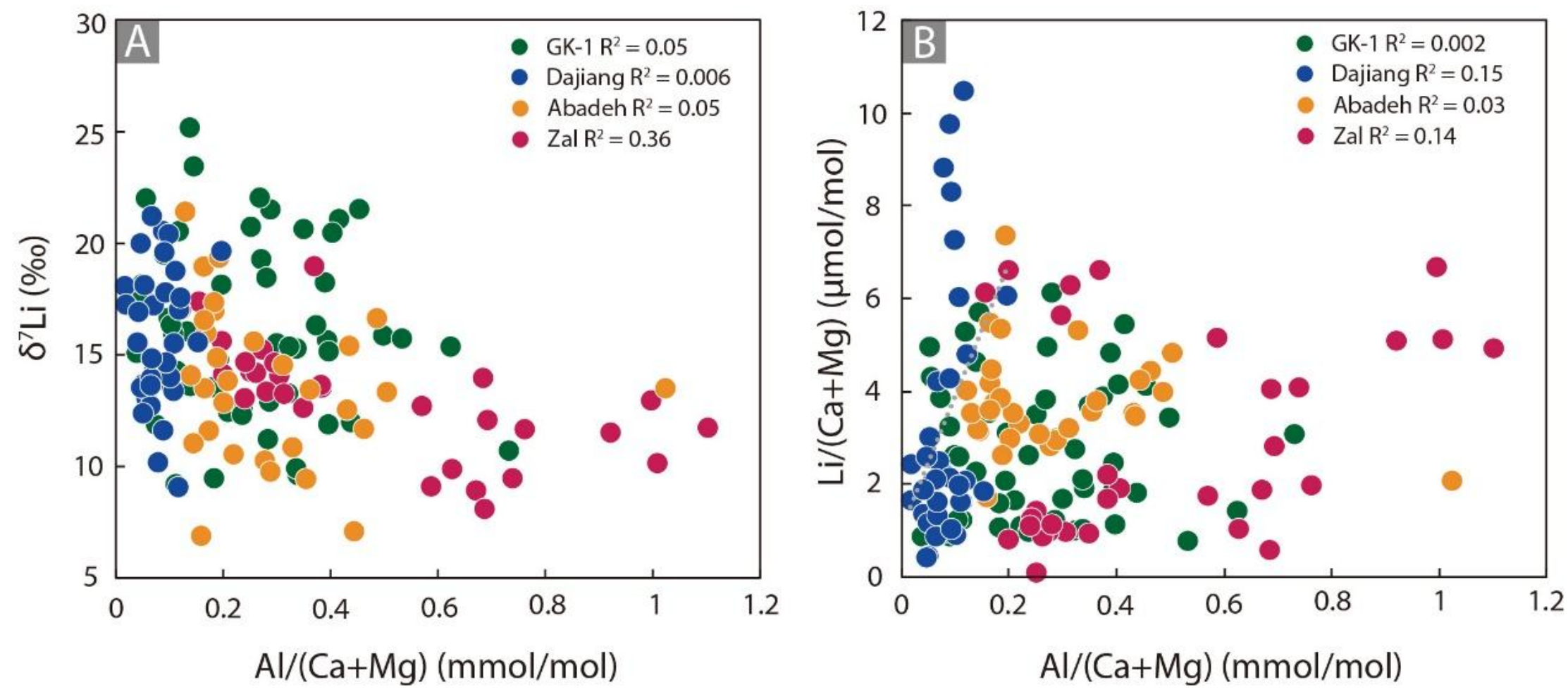

Figure S2. Evaluation of detrital contamination. The cross-plots of δ^7Li and Li/(Ca+Mg) versus Al/(Ca+Mg) of the analyzed carbonates as indicators for potential detrital contaminations during sample digestion.

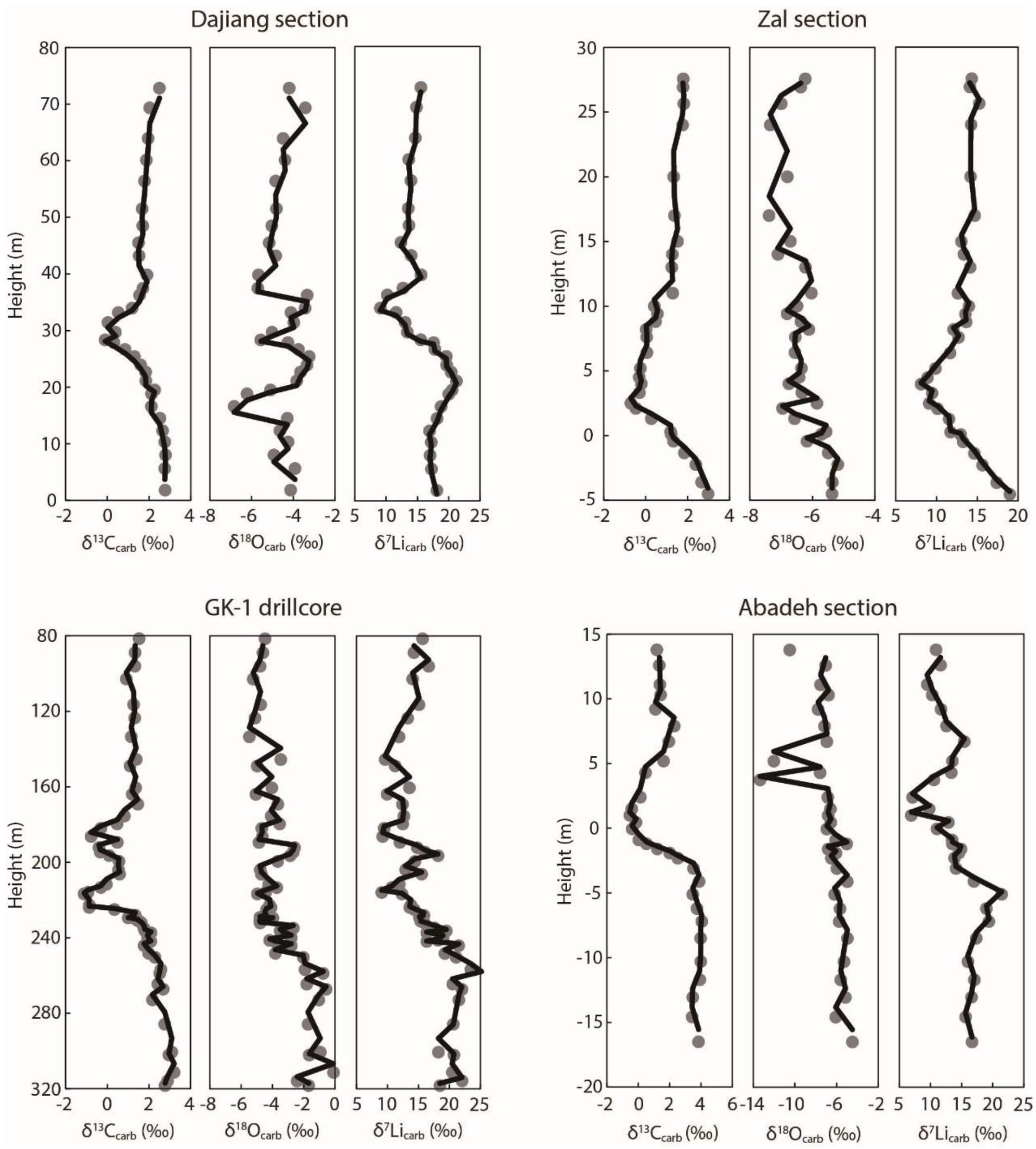

Figure S3. Chemostratigraphic profiles of carbon, oxygen, and lithium isotopes.

Variations in carbonate $\delta^{13}C$, $\delta^{18}O$ and $\delta^{7}Li$ signatures in the Dajiang section (South China), Zal and Abadeh sections (Iran), and GK-1 drillcore (the western Austria).

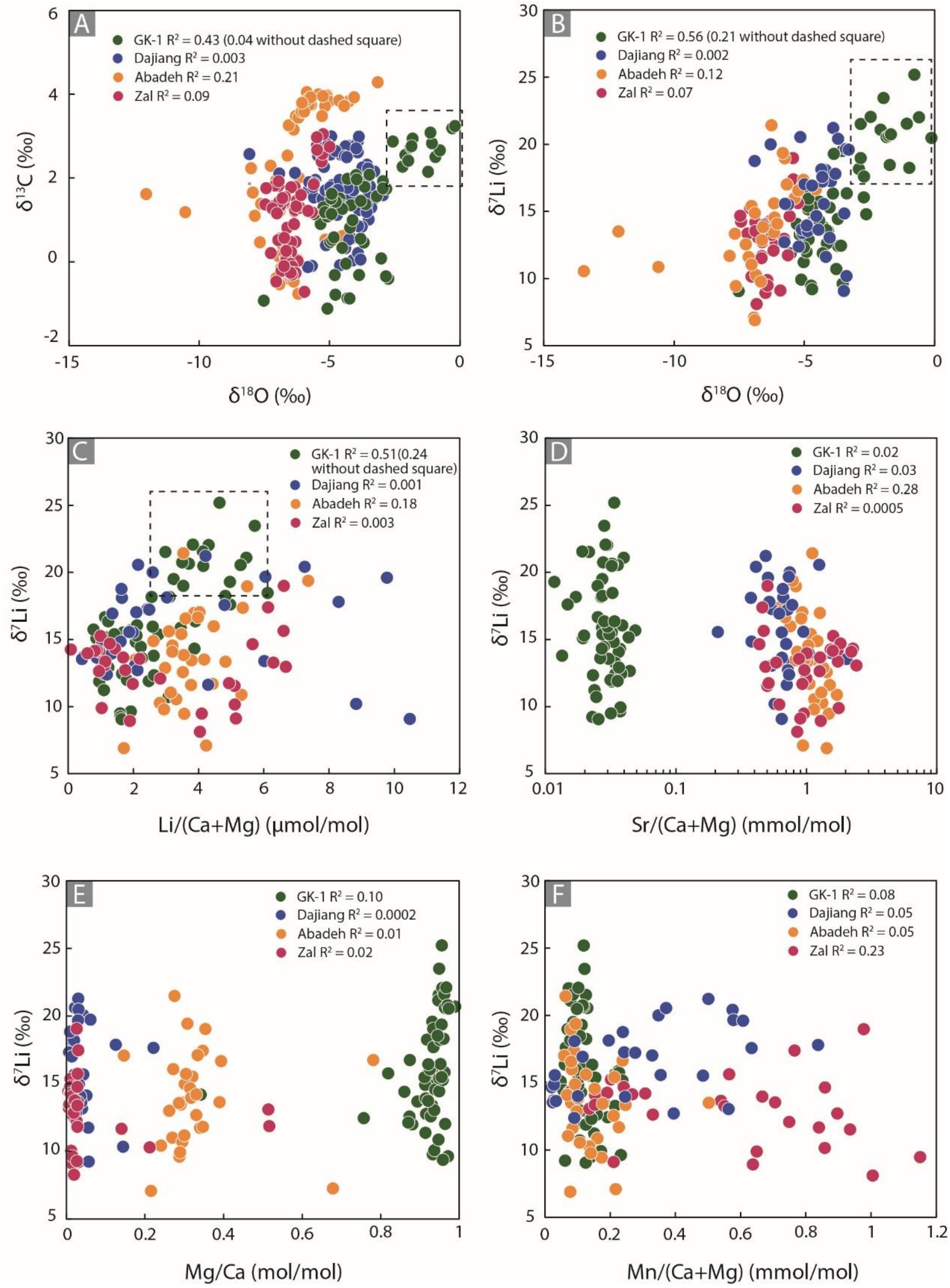

Figure S4. Evaluation of carbonate diagenesis. $\delta^{13}C$ versus $\delta^{18}O$ **(A)**, and $\delta^{7}Li$ versus **(B)** $\delta^{18}O$, **(C)** Li/(Ca+Mg), **(D)** Sr/(Ca+Mg), **(E)** Mg/Ca, **(F)** Mn/(Ca+Mg) for the analyzed carbonates, in order to evaluate the mineralogical and diagenetic effects on carbonate Li isotope records. R^2 values represent the coefficient of determination of the covariation in each section.

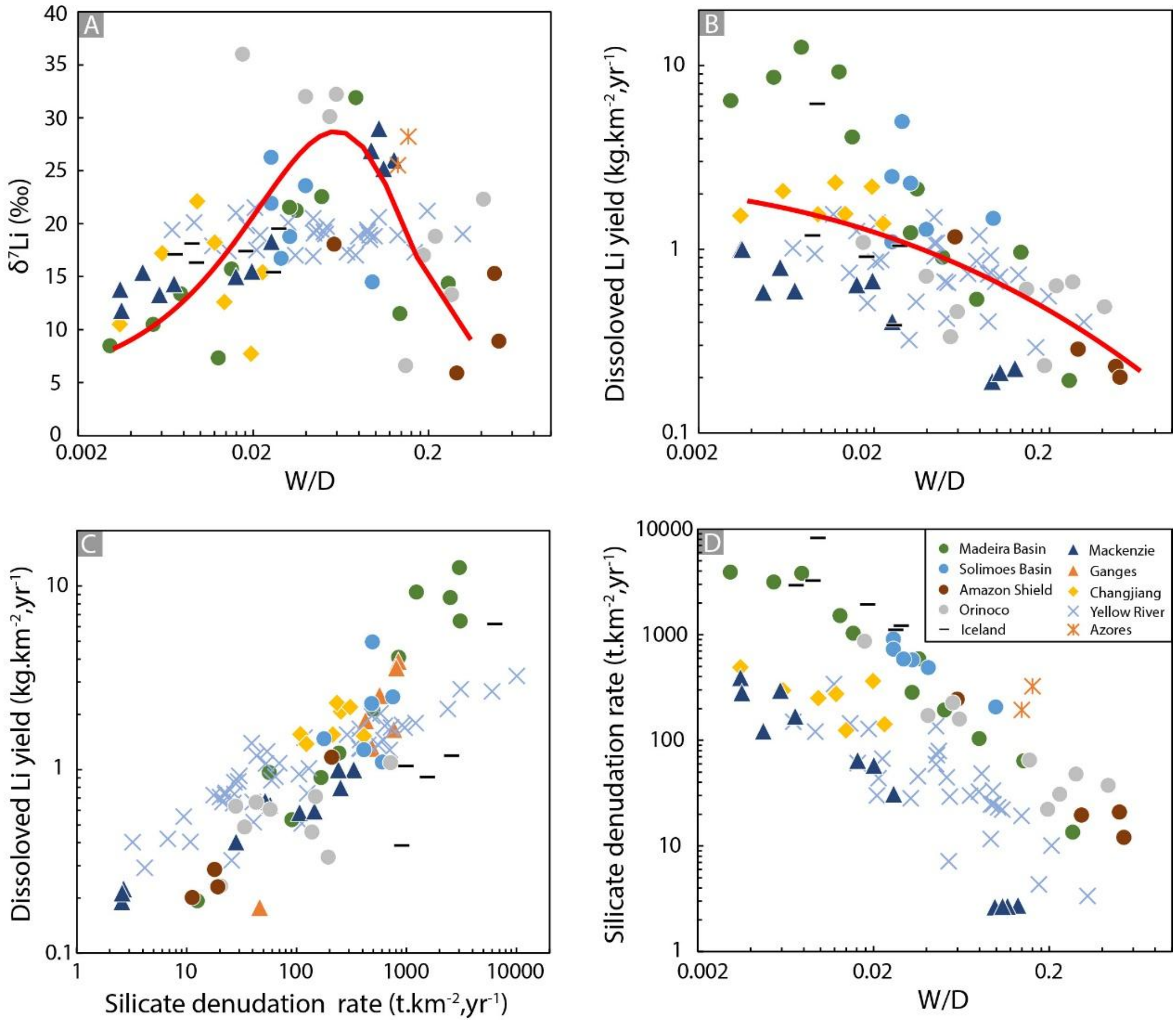

Figure S5. Modern riverine Li isotope framework. Variations of riverine weathering proxies as a function of weathering intensity (W/D): W/D versus **(A)** δ^7Li, **(B)** Li yield, and **(D)** silicate denudation rate, and **(C)** silicate denudation rate versus Li yield. In panels **(A)** and **(B)**, the red curves represent the modeled results of δ^7Li and Li flux, which are used to calculate global seawater δ^7Li evolution (see Supplementary text for details). Primary data from modern riverine systems (*49, 112*).

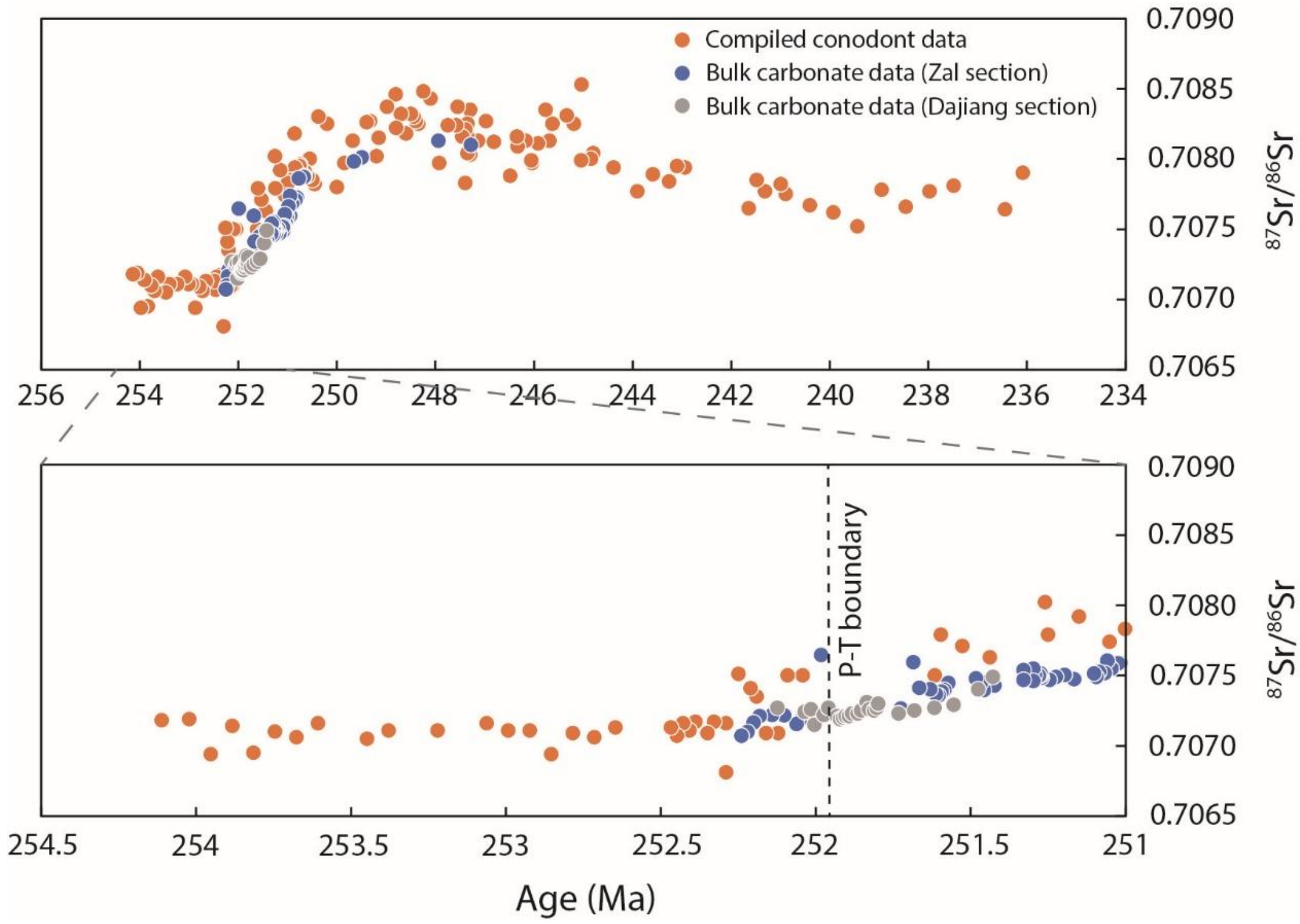

Figure S6. Compiled seawater radiogenic strontium isotope records. Integrated seawater $^{87}Sr/^{86}Sr$ curve during the P-Tr transition derived from conodonts (*26, 83*), bulk carbonate records (*27, 113*).

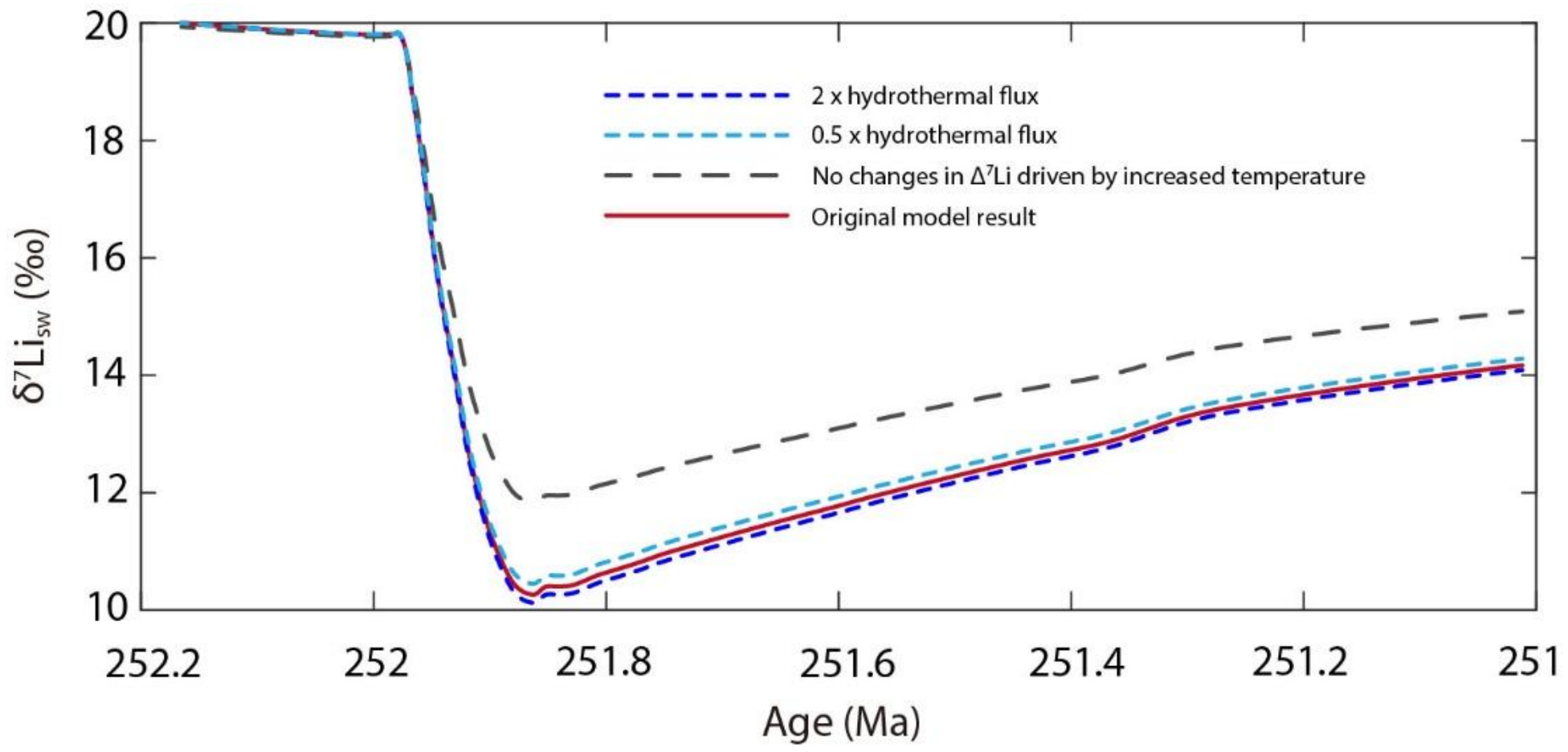

Figure S7. Sensitivity tests of global marine lithium isotope mass balance model. Evaluation on global marine Li isotope mass balance for changes in hydrothermal fluxes and isotopic fractionations between Li sinks and seawater.

S3. Supplementary Table S1. Model parameters used in global marine Li mass balance model

Parameter	Description	Value	Unit	Reference
F_{riv0}	Modern riverine Li flux to ocean	10×10^9	mol/yr	(*114*)
$F_{riv\text{-}P}$	Estimated late-Permian value	12×10^9	mol/yr	This study
F_{hyd0}	Modern High-T hydrothermal Li flux to ocean	5.2×10^9	mol/yr	(*48*)
F_{AOC}	Li output flux to altered oceanic crust	$K_{AOC} \bullet [Li]$	mol/yr	This study
F_{MAAC}	Li output flux to marine authigenic clay	$K_{MAAC} \bullet [Li]$	mol/yr	This study
K_{MAAC}	Calculated Li MAAC output constant	2.0	10^{-6}/yr	This study
K_{AOC}	Calculated Li AOC output constant	1.8	10^{-6}/yr	This study
δ^7Li_{riv0}	Modern riverine δ^7Li value	+23	‰	(*115*)
$\delta^7Li_{riv\text{-}P}$	Estimated late-Permian value	+20	‰	This study
δ^7Li_{hdy0}	Modern riverine δ^7Li value	+6.3	‰	(*48*)
$\Delta^7Li_{AOC\text{-}P}$	Li fractionation between altered oceanic crust and late-Permian seawater	-10	‰	(*97*)
$\Delta^7Li_{MAAC\text{-}P}$	Li fractionation between marine authigenic clay and late-Permian seawater	-5	‰	(*97*)
δ^7Li_{sw0}	Modern seawater δ^7Li value	+30.9	‰	(*114*)
$\delta^7Li_{sw\text{-}P}$	Estimated late-Permian value	21	‰	This study
$[Li]_{sw0}$	Modern seawater Li concentration	2.6	µmol/L	(*114*)
N_{Li0}	Modern seawater Li reservoir	18.2×10^{15}	mol	(*114*)
$N_{Li\text{-}P}$	Estimated late-Permian value	20.0×10^{15}	mol	This study
δ^7Li_{rock}	Average unweathered bedrock	+1.0	‰	(*116*)
a	Parameterized constant	4.5		(*107*)
b	Parameterized constant	0.06		(*107*)

S4. Supplementary Table S2. Geochemical data in this study

The geochemical data supporting the findings of this study are available within the paper and are publicly available at Zenodo with a https://zenodo.org/records/17337223 (doi: 10.5281/zenodo.17337223).

REFERENCES

1. M. J. Benton, The origins of modern biodiversity on land. *Philos. Trans. R. Soc. B Biol. Sci.* **365**, 3667–3679 (2010).

2. S.-Z. Shen, J. L. Crowley, Y. Wang, S. A. Bowring, D. H. Erwin, P. M. Sadler, C.-Q. Cao, D. H. Rothman, C. M. Henderson, J. Ramezani, H. Zhang, Y. Shen, X.-D. Wang, W. Wang, L. Mu, W.-Z. Li, Y.-G. Tang, X.-L. Liu, L.-J. Liu, Y. Zeng, Y.-F. Jiang, Y.-G. Jin, Calibrating the end-Permian mass extinction. *Science* **334**, 1367–1372 (2011).

3. J. L. Payne, D. J. Lehrmann, J. Wei, M. J. Orchard, D. P. Schrag, A. H. Knoll, Large perturbations of the carbon cycle during recovery from the end-Permian extinction. *Science* **305**, 506–509 (2004).

4. T. J. Algeo, Z. Q. Chen, M. L. Fraiser, R. J. Twitchett, Terrestrial-marine teleconnections in the collapse and rebuilding of Early Triassic marine ecosystems. *Palaeogeogr. Palaeoclimatol. Palaeoecol.* **308**, 1–11 (2011).

5. Z.-Q. Chen, M. J. Benton, The timing and pattern of biotic recovery following the end-Permian mass extinction. *Nat. Geosci.* **5**, 375–383 (2012).

6. M. M. Joachimski, X. Lai, S. Shen, H. Jiang, G. Luo, B. Chen, J. Chen, Y. Sun, Climate warming in the latest Permian and the Permian-Triassic mass extinction. *Geology* **40**, 195–198 (2012).

7. K. V. Lau, K. Maher, D. Altiner, B. M. Kelley, L. R. Kump, D. J. Lehrmann, J. C. Silva-Tamayo, K. L. Weaver, M. Yu, J. L. Payne, Marine anoxia and delayed Earth system recovery after the end-Permian extinction. *Proc. Natl. Acad. Sci. U.S.A.* **113**, 2360–2365 (2016).

8. H. Jurikova, M. Gutjahr, K. Wallmann, S. Flögel, V. Liebetrau, R. Posenato, L. Angiolini, C. Garbelli, U. Brand, M. Wiedenbeck, A. Eisenhauer, Permian-Triassic mass extinction pulses driven by major marine carbon cycle perturbations. *Nat. Geosci.* **13**, 745–750 (2020).

9. Y. Sun, A. Farnsworth, M. M. Joachimski, P. B. Wignall, L. Krystyn, D. P. G. Bond, D. C. G. Ravida, P. J. Valdes, Mega El Nino instigated the end-Permian mass extinction. *Science* **385**, 1189–1195 (2024).

10. S. D. Burgess, J. D. Muirhead, S. A. Bowring, Initial pulse of Siberian Traps sills as the trigger of the end-Permian mass extinction. *Nat. Commun.* **8**, 164 (2017).

11. H. Zhang, F. Zhang, J. B. Chen, D. H. Erwin, D. D. Syverson, P. Ni, M. Rampino, Z. Chi, Y. F. Cai, L. Xiang, W. Q. Li, S. A. Liu, R. C. Wang, X. D. Wang, Z. Feng, H. M. Li, T. Zhang, H. M. Cai, W. Zheng, Y. Cui, X. K. Zhu, Z. Q. Hou, F. Y. Wu, Y. G. Xu, N. Planavsky, S. Z. Shen, Felsic volcanism as a factor driving the end-Permian mass extinction. *Sci. Adv.* **7**, eabh1390 (2021).

12. Y. Wu, D. Chu, J. Tong, H. Song, J. Dal Corso, P. B. Wignall, H. Song, Y. Du, Y. Cui, Six-fold increase of atmospheric pCO_2 during the Permian-Triassic mass extinction. *Nat. Commun.* **12**, 2137 (2021).

13. M. M. Joachimski, J. Muller, T. M. Gallagher, G. Mathes, D. L. Chu, F. Mouraviev, V. Silantiev, Y. D. Sun, J. N. Tong, Five million years of high atmospheric CO_2 in the aftermath of the Permian-Triassic mass extinction. *Geology* **50**, 650–654 (2022).

14. Y. Sun, M. M. Joachimski, P. B. Wignall, C. Yan, Y. Chen, H. Jiang, L. Wang, X. Lai, Lethally hot temperatures during the Early Triassic greenhouse. *Science* **338**, 366–370 (2012).

15. M. W. Broadley, P. H. Barry, C. J. Ballentine, L. A. Taylor, R. Burgess, End-Permian extinction amplified by plume-induced release of recycled lithospheric volatiles. *Nat. Geosci.* **11**, 682–687 (2018).

16. M. J. Benton, A. J. Newell, Impacts of global warming on Permo-Triassic terrestrial ecosystems. *Gondw. Res.* **25**, 1308–1337 (2014).

17. J. Dal Corso, H. Song, S. Callegaro, D. Chu, Y. Sun, J. Hilton, S. E. Grasby, M. M. Joachimski, P. B. Wignall, Environmental crises at the Permian-Triassic mass extinction. *Nat. Rev. Earth Environ.* **3**, 197–214 (2022).

18. Y. Cui, M. Li, E. E. van Soelen, F. Peterse, W. M. Kürschner, Massive and rapid predominantly volcanic CO_2 emission during the end-Permian mass extinction. *Proc. Natl. Acad. Sci. U.S.A.* **118**, e2014701118 (2021).

19. G. L. Foster, P. Hull, D. J. Lunt, J. C. Zachos, Placing our current 'hyperthermal' in the context of rapid climate change in our geological past. *Philos. Trans. A Math. Phys. Eng. Sci.* **376**, 20170086 (2018).

20. J. C. G. Walker, P. B. Hays, J. F. Kasting, A negative feedback mechanism for the long-term stabilization of Earth's surface temperature. *J. Geophys. Res.* **86**, 9776–9782 (1981).

21. R. A. Berner, A. C. Lasaga, R. M. Garrels, The carbonate-silicate geochemical cycle and its effect on atmospheric carbon dioxide over the past 100 million years. *Am. J. Sci.* **283**, 641–683 (1983).

22. L. R. Kump, S. L. Brantley, M. A. Arthur, Chemical weathering, atmospheric CO_2, and climate. *Annu. Rev. Earth Planet. Sci.* **28**, 611–667 (2000).

23. L. R. Kump, Prolonged Late Permian-Early Triassic hyperthermal: Failure of climate regulation? *Philos. Trans. A. Math. Phys. Eng. Sci.* **376**, 20170078 (2018).

24. T. J. Algeo, R. J. Twitchett, Anomalous Early Triassic sediment fluxes due to elevated weathering rates and their biological consequences. *Geology* **38**, 1023–1026 (2010).

25. G. Xu, J. Shen, T. J. Algeo, J. Yu, Q. Feng, T. D. Frank, C. R. Fielding, J. Yan, J.-F. Deconink, Y. Lei, Limited change in silicate chemical weathering intensity during the Permian-Triassic transition indicates ineffective climate regulation by weathering feedbacks. *Earth Planet. Sci. Lett.* **616**, 118235 (2023).

26. H. Song, P. B. Wignall, J. Tong, H. Song, J. Chen, D. Chu, L. Tian, M. Luo, K. Zong, Y. Chen, X. Lai, K. Zhang, H. Wang, Integrated Sr isotope variations and global environmental changes through the Late Permian to early Late Triassic. *Earth Planet. Sci. Lett.* **424**, 140–147 (2015).

27. A. R. C. Sedlacek, M. R. Saltzman, T. J. Algeo, M. Horacek, R. Brandner, K. Foland, R. F. Denniston, $^{87}Sr/^{86}Sr$ stratigraphy from the Early Triassic of Zal, Iran: Linking temperature to weathering rates and the tempo of ecosystem recovery. *Geology* **42**, 779–782 (2014).

28. P. A. E. Pogge von Strandmann, H. C. Jenkyns, R. G. Woodfine, Lithium isotope evidence for enhanced weathering during Oceanic Anoxic Event 2. *Nat. Geosci.* **6**, 668–672 (2013).

29. M. Dellinger, D. S. Hardisty, N. J. Planavsky, B. C. Gill, B. Kalderon-Asael, D. Asael, T. Croissant, P. K. Swart, A. J. West, The effects of diagenesis on lithium isotope ratios of shallow marine carbonates. *Am. J. Sci.* **320**, 150–184 (2020).

30. C. S. Marriott, G. M. Henderson, R. Crompton, M. Staubwasser, S. Shaw, Effect of mineralogy, salinity, and temperature on Li/Ca and Li isotope composition of calcium carbonate. *Chem. Geol.* **212**, 5–15 (2004).

31. A. Füger, M. Kuessner, C. Rollion-Bard, A. Leis, T. Magna, M. Dietzel, V. Mavromatis, Effect of growth rate and pH on Li isotope fractionation during its incorporation in calcite. *Geochim. Cosmochim. Acta* **323**, 276–290 (2022).

32. J. G. Murphy, A.-S. C. Ahm, P. K. Swart, J. A. Higgins, Reconstructing the lithium isotopic composition (δ^7Li) of seawater from shallow marine carbonate sediments. *Geochim. Cosmochim. Acta* **337**, 140–154 (2022).

33. G.-Y. Wei, F. Zhang, Y.-S. Yin, Y.-B. Lin, P. A. E. Pogge von Strandmann, M. Cao, N. Li, G. Xiong, X. Chen, C. Fan, C. Xu, F. Tan, X. Zhang, H. Yang, H.-F. Ling, S.-Z. Shen, A 13 million-year record of Li isotope compositions in island carbonates: Constraints on bulk inorganic carbonate as a global seawater Li isotope archive. *Geochim. Cosmochim. Acta* **344**, 59–72 (2023).

34. E. Heydari, W. J. Wade, J. Hassanzadeh, Diagenetic origin of carbon and oxygen isotope compositions of Permian-Triassic boundary strata. *Sediment. Geol.* **143**, 191–197 (2001).

35. H. Yin, H. Jiang, W. Xia, Q. Feng, N. Zhang, J. Shen, The end-Permian regression in South China and its implication on mass extinction. *Earth Sci. Rev.* **137**, 19–33 (2014).

36. G.-Y. Wei, F. Zhang, Pristine or altered, what can early diagenesis tell us in shallow-water carbonates? *Earth Planet. Sci. Lett.* **641**, 118806 (2024).

37. S. D. Burgess, S. Bowring, S. Z. Shen, High-precision timeline for Earth's most severe extinction. *Proc. Natl. Acad. Sci. U.S.A.* **111**, 3316–3321 (2014).

38. Q. Wu, H. Zhang, J. Ramezani, F.-F. Zhang, D. H. Erwin, Z. Feng, L.-Y. Shao, Y.-F. Cai, S.-H. Zhang, Y.-G. Xu, S.-Z. Shen, The terrestrial end-Permian mass extinction in the paleotropics postdates the marine extinction. *Sci. Adv.* **10**, eadi7284 (2024).

39. S.-Z. Shen, C.-Q. Cao, H. Zhang, S. A. Bowring, C. M. Henderson, J. L. Payne, V. I. Davydov, B. Chen, D.-X. Yuan, Y.-C. Zhang, W. Wang, Q.-F. Zheng, High-resolution $\delta^{13}C_{carb}$ chemostratigraphy from latest Guadalupian through earliest Triassic in South China and Iran. *Earth Planet. Sci. Lett.* **375**, 156–165 (2013).

40. H. Zhang, C.-Q. Cao, X.-L. Liu, L. Mu, Q.-F. Zheng, F. Liu, L. Xiang, L.-J. Liu, S.-Z. Shen, The terrestrial end-Permian mass extinction in South China. *Palaeogeogr. Palaeoclimatol. Palaeoecol.* **448**, 108–124 (2016).

41. P. A. E. Pogge von Strandmann, M. T. Jones, A. J. West, M. J. Murphy, E. W. Stokke, G. Tarbuck, D. J. Wilson, C. R. Pearce, D. N. Schmidt, Lithium isotope evidence for enhanced weathering and erosion during the Paleocene-Eocene Thermal Maximum. *Sci. Adv.* **7**, eabh4224 (2021).

42. C. Cao, C. P. Bataille, H. Song, M. R. Saltzman, K. Tierney Cramer, H. Wu, C. Korte, Z. Zhang, X.-M. Liu, Persistent late Permian to Early Triassic warmth linked to enhanced reverse weathering. *Nat. Geosci.* **15**, 832–838 (2022).

43. S. Rauzi, W. J. Foster, S. Takahashi, R. S. Hori, B. J. Beaty, L. G. Tarhan, T. Isson, Lithium isotopic evidence for enhanced reverse weathering during the Early Triassic warm period. *Proc. Natl. Acad. Sci. U.S.A.* **121**, e2318860121 (2024).

44. T. Algeo, C. M. Henderson, B. Ellwood, H. Rowe, E. Elswick, S. Bates, T. Lyons, J. C. Hower, C. Smith, B. Maynard, L. E. Hays, R. E. Summons, J. Fulton, K. H. Freeman, Evidence for a

diachronous Late Permian marine crisis from the Canadian Arctic region. *Geol. Soc. Am. Bull.* **124**, 1424–1448 (2012).

45. W. J. Foster, G. Asatryan, S. Rauzi, J. P. Botting, S. Z. Buchwald, D. B. Lazarus, T. Isson, J. Renaudie, W. Kiessling, Response of siliceous marine organisms to the Permian-Triassic climate crisis based on new findings from central Spitsbergen, Svalbard. *Paleoceanogr. Paleoclimatol.* **38**, e2023PA004766 (2023).

46. J. Zheng, X. Jin, H. Huang, Z. Yan, Died out at the end of Permian or extended into the Triassic?—The tale of the albaillellarians (radiolarians) and detrital zircons of the Nanpihe bridge section in the Changning-Menglian belt, Western Yunnan, China, *Palaeogeogr. Palaeoclimatol. Palaeoecol.* **673**, 112988 (2025).

47. G. Li, A. J. West, Evolution of Cenozoic seawater lithium isotopes: Coupling of global denudation regime and shifting seawater sinks. *Earth Planet. Sci. Lett.* **401**, 284–293 (2014).

48. L. A. Coogan, K. M. Gillis, M. Pope, J. Spence, The role of low-temperature (off-axis) alteration of the oceanic crust in the global Li-cycle: Insights from the Troodos ophiolite. *Geochim. Cosmochim. Acta* **203**, 201–215 (2017).

49. M. Dellinger, J. Gaillardet, J. Bouchez, D. Calmels, P. Louvat, A. Dosseto, C. Gorge, L. Alanoca, L. Maurice, Riverine Li isotope fractionation in the Amazon River basin controlled by the weathering regimes. *Geochim. Cosmochim. Acta* **164**, 71–93 (2015).

50. P. A. E. Pogge von Strandmann, M. Dellinger, A. J. West, *Lithium Isotopes: A Tracer of Past and Present Silicate Weathering* (Cambridge Univ. Press, 2021).

51. G.-Y. Wei, W. Wei, D. Wang, T. Li, X. Yang, G. A. Shields, F. Zhang, G. Li, T. Chen, T. Yang, H.-F. Ling, Enhanced chemical weathering triggered an expansion of euxinic seawater in the aftermath of the Sturtian glaciation. *Earth Planet. Sci. Lett.* **539**, 116244 (2020).

52. G.-Y. Wei, M. Zhao, E. A. Sperling, R. R. Gaines, B. Kalderon-Asael, J. Shen, C. Li, F. Zhang, G. Li, C. Zhou, C. Cai, D. Chen, K.-Q. Xiao, L. Jiang, H.-F. Ling, N. J. Planavsky, L. G. Tarhan,

Lithium isotopic constraints on the evolution of continental clay mineral factory and marine oxygenation in the earliest Paleozoic Era. *Sci. Adv.* **10**, eadk2152 (2024).

53. R.-H. Ye, F. Zhang, G.-Y. Wei, J. Chen, Z. Feng, S.-Z. Shen, Lithium isotopes track changes in continental weathering regimes across the end-Permian mass extinction in Southwest China. *Earth Planet. Sci. Lett.* **647**, 119045 (2024).

54. M. Dellinger, J. Gaillardet, J. Bouchez, D. Calmels, V. Galy, R. G. Hilton, P. Louvat, C. France-Lanord, Lithium isotopes in large rivers reveal the cannibalistic nature of modern continental weathering and erosion. *Earth Planet. Sci. Lett.* **401**, 359–372 (2014).

55. C. R. Fielding, T. D. Frank, S. McLoughlin, V. Vajda, C. Mays, A. P. Tevyaw, A. Winguth, C. Winguth, R. S. Nicoll, M. Bocking, J. L. Crowley, Age and pattern of the southern high-latitude continental end-Permian extinction constrained by multiproxy analysis. *Nat. Commun.* **10**, 385 (2019).

56. R. A. Gastaldo, S. L. Kamo, J. Neveling, J. W. Geissman, C. V. Looy, A. M. Martini, The base of the Lystrosaurus Assemblage Zone, Karoo Basin, predates the end-Permian marine extinction. *Nat. Commun.* **11**, 1428 (2020).

57. W. Guo, J. Tong, Q. He, M. W. Hounslow, H. Song, J. Dal Corso, P. B. Wignall, J. Ramezani, L. Tian, D. Chu, Late Permian-Middle Triassic magnetostratigraphy in North China and its implications for terrestrial-marine correlations. *Earth Planet. Sci. Lett.* **585**, 117519 (2022).

58. A. J. West, Thickness of the chemical weathering zone and implications for erosional and climatic drivers of weathering and for carbon-cycle feedbacks. *Geology* **40**, 811–814 (2012).

59. Z. Xu, J. Hilton, J. Yu, P. B. Wignall, H. Yin, Q. Xue, W. Ran, H. Li, J. Shen, F. Meng, End Permian to Middle Triassic plant species richness and abundance patterns in South China: Coevolution of plants and the environment through the Permian-Triassic transition. *Earth Sci. Rev.* **232**, 104136 (2022).

60. V. I. Davydov, E. V. Karasev, N. G. Nurgalieva, M. D. Schmitz, I. V. Budnikov, A. S. Biakov, D. M. Kuzina, V. V. Silantiev, M. N. Urazaeva, V. V. Zharinova, S. O. Zorina, B. Gareev, D. V.

Vasilenko, Climate and biotic evolution during the Permian-Triassic transition in the temperate Northern Hemisphere, Kuznetsk Basin, Siberia, Russia, *Palaeogeogr. Palaeoclimatol. Palaeoecol.* **573**, 103438 (2021).

61. Y. Eshet, M. R. Rampino, H. Visscher, Fungal event and palynological record of ecological crisis and recovery across the Permian-Triassic boundary. *Geology* **23**, 967–970 (1995).

62. J. Wu, D. Chu, G. Luo, P. B. Wignall, T. J. Algeo, S. Xie, Stepwise deforestation during the Permian-Triassic boundary crisis linked to rising temperatures. *Earth Planet. Sci. Lett.* **620**, 118350 (2023).

63. L. Grauvogel-Stamm, S. R. Ash, Recovery of the Triassic land flora from the end-Permian life crisis. *C. R. Palevol* **4**, 593–608 (2005).

64. C. V. Looy, W. A. Brugman, D. L. Dilcher, H. Visscher, The delayed resurgence of equatorial forests after the Permian-Triassic ecologic crisis. *Proc. Natl. Acad. Sci. U.S.A.* **96**, 13857–13862 (1999).

65. G. J. Retallack, J. J. Veevers, R. Morante, Global coal gap between Permian-Triassic extinction and Middle Triassic recovery of peat-forming plants. *Geol. Soc. Am. Bull.* **108**, 195–207 (1996).

66. C. Baars, T. Hefin Jones, D. Edwards, Microcosm studies of the role of land plants in elevating soil carbon dioxide and chemical weathering. *Global Biogeochem. Cycles* **22**, GB3019 (2008).

67. J. Quirk, J. R. Leake, D. A. Johnson, L. L. Taylor, L. Saccone, D. J. Beerling, Constraining the role of early land plants in Palaeozoic weathering and global cooling. *Proc. Biol. Sci.* **282**, 20151115 (2015).

68. M. Ford Cochran, R. A. Berner, Promotion of chemical weathering by higher plants: Field observations on Hawaiian basalts. *Chem. Geol.* **132**, 71–77 (1996).

69. S. Porder, How plants enhance weathering and how weathering is important to plants. *Elements* **15**, 241–246 (2019).

70. W. J. McMahon, N. S. Davies, Evolution of alluvial mudrock forced by early land plants. *Science* **359**, 1022–1024 (2018).

71. Y. Godderis, C. Roelandt, J. Schott, M. C. Pierret, L. M. Francois, Towards an integrated model of weathering, climate, and biospheric processes. *Rev. Mineral. Geochem.* **70**, 411–434 (2009).

72. P. Maffre, Y. Godderis, A. Pohl, Y. Donnadieu, S. Carretier, G. Le Hir, The complex response of continental silicate rock weathering to the colonization of the continents by vascular plants in the Devonian. *Am. J. Sci.* **322**, 461–492 (2022).

73. V. Vanacker, F. von Blanckenburg, G. Govers, A. Molina, J. Poesen, J. Deckers, P. Kubik, Restoring dense vegetation can slow mountain erosion to near natural benchmark levels. *Geology* **35**, 303–306 (2007).

74. A. Carriere, C. Le Bouteiller, G. E. Tucker, S. Klotz, M. Naaim, Impact of vegetation on erosion: Insights from the calibration and test of a landscape evolution model in alpine badland catchments. *Earth Surf. Process. Landf.* **45**, 1085–1099 (2020).

75. X. Liu, D. J. Wilson, K. W. Burton, B. D. Sigurdsson, J. C. Bos, W. T. Fraser, P. A. E. Pogge von Strandmann, Rapid and significant lithium isotope response to afforestation in Icelandic topsoils. *Catena* **254**, 108967 (2025).

76. X. Liu, A. J. Krause, D. J. Wilson, W. T. Fraser, M. M. Joachimski, U. Brand, A. L. Stigall, W. Qie, B. Chen, X. Yang, P. A. E. Pogge von Strandmann, Lithium isotope evidence shows Devonian afforestation may have significantly altered the global silicate weathering regime. *Geochim. Cosmochim. Acta* **396**, 107–121 (2025).

77. Y. Goddéris, Y. Donnadieu, G. Le Hir, V. Lefebvre, E. Nardin, The role of palaeogeography in the Phanerozoic history of atmospheric CO_2 and climate. *Earth Sci. Rev.* **128**, 122–138 (2014).

78. B. J. W. Mills, Y. Donnadieu, Y. Godderis, Spatial continuous integration of Phanerozoic global biogeochemistry and climate. *Gondw. Res.* **100**, 73–86 (2021).

79. Y. Wu, Y. Cui, D. Chu, H. Song, J. Tong, J. Dal Corso, A. Ridgwell, Volcanic CO_2 degassing postdates thermogenic carbon emission during the end-Permian mass extinction. *Sci. Adv.* **9**, eabq4082 (2023).

80. Z. Xu, J. Yu, H. Yin, A. S. Merdith, J. Hilton, B. J. Allen, K. Gurung, P. B. Wignall, A. M. Dunhill, J. Shen, D. Schwartzman, Y. Godderis, Y. Donnadieu, Y. Wang, Y. Zhang, S. W. Poulton, B. J. W. Mills, Early Triassic super-greenhouse climate driven by vegetation collapse. *Nat. Commun.* **16**, 5400 (2025).

81. M. E. Clapham, P. R. Renne, Flood basalts and mass extinctions. *Annu. Rev. Earth Planet. Sci.* **47**, 275–303 (2019).

82. C. Korte, H. W. Kozur, P. Bruckschen, J. Veizer, Strontium isotope evolution of Late Permian and Triassic seawater. *Geochim. Cosmochim. Acta* **67**, 47–62 (2003).

83. F. Ö. Dudás, D.-X. Yuan, S.-Z. Shen, S. A. Bowring, A conodont-based revision of the $^{87}Sr/^{86}Sr$ seawater curve across the Permian-Triassic boundary. *Palaeogeogr. Palaeoclimatol. Palaeoecol.* **470**, 40–53 (2017).

84. L. Guo, S. Xiong, B. J. W. Mills, T. Isson, S. Yang, J. Cui, Y. Wang, L. Jiang, Z. Xu, C. Cai, Y. Deng, G. Wei, M. Zhao, Acceleration of phosphorus weathering under warm climates. *Sci. Adv.* **10**, eadm7773 (2024).

85. M. Y. Zhao, B. J. W. Mills, S. W. Poulton, B. Wan, K. Q. Xiao, L. C. Guo, Z. T. Guo, Drivers of the global phosphorus cycle over geological time. *Nat. Rev. Earth Environ.* **5**, 873–889 (2024).

86. T. M. Lenton, M. Crouch, M. Johnson, N. Pires, L. Dolan, First plants cooled the Ordovician. *Nat. Geosci.* **5**, 86–89 (2012).

87. J. Dal Corso, B. J. W. Mills, D. Chu, R. J. Newton, T. A. Mather, W. Shu, Y. Wu, J. Tong, P. B. Wignall, Permo-Triassic boundary carbon and mercury cycling linked to terrestrial ecosystem collapse. *Nat. Commun.* **11**, 2962 (2020).

88. T. Zhao, S. Xu, F. Hao, Differential adsorption of clay minerals: Implications for organic matter enrichment. *Earth Sci. Rev.* **246**, 104598 (2023).

89. G.-Y. Wei, A. Pohl, S. Jiang, H. Zhang, W. Wang, P. A. E. Pogge von Strandmann, P. Maffre, G. Xiong, S.-Z. Shen, F. Zhang, Changes in continental weathering regimes inhibited global marine deoxygenation during the Paleocene-Eocene thermal maximum. *Nat. Commun.* **16**, 9163 (2025).

90. Z.-H. Li, T. M. Lenton, F.-F. Zhang, Z.-Q. Chen, S. J. Daines, Earth system instability amplified biogeochemical oscillations following the end-Permian mass extinction. *Nat. Commun.* **16**, 3703 (2025).

91. C. R. Scotese, An atlas of Phanerozoic paleogeographic maps: The seas come in and the seas go out. *Annu. Rev. Earth Planet. Sci.* **49**, 679–728 (2021).

92. J. Chen, T. W. Beatty, C. M. Henderson, H. Rowe, Conodont biostratigraphy across the Permian-Triassic boundary at the Dawen section, Great Bank of Guizhou, Guizhou Province, South China: Implications for the Late Permian extinction and correlation with Meishan. *J. Asian Earth Sci.* **36**, 442–458 (2009).

93. M. Horacek, S. Richoz, R. Brandner, L. Krystyn, C. Spötl, Evidence for recurrent changes in Lower Triassic oceanic circulation of the Tethys: The $\delta^{13}C$ record from marine sections in Iran. *Palaeogeogr. Palaeoclimatol. Palaeoecol.* **252**, 355–369 (2007).

94. S. Arefifard, A. Baud, Depositional environment and sequence stratigraphy architecture of continuous Upper Permian and Lowermost Triassic deep marine deposits in NW and SW Iran. *Palaeogeogr. Palaeoclimatol. Palaeoecol.* **603**, 111187 (2022).

95. W. T. Holser, H.-P. Schönlaub, M. Attrep, K. Boeckelmann, P. Klein, M. Magaritz, C. J. Orth, A. Fenninger, C. Jenny, M. Kralik, H. Mauritsch, E. Pak, J.-M. Schramm, K. Stattegger, R. Schmöller, A unique geochemical record at the Permian/Triassic boundary. *Nature* **337**, 39–44 (1989).

96. M. R. Rampino, A. Prokoph, A. Adler, Tempo of the end-Permian event: High-resolution cyclostratigraphy at the Permian-Triassic boundary. *Geology* **28**, 643–646 (2000).

97. B. Kalderon-Asael, J. A. R. Katchinoff, N. J. Planavsky, A. V. S. Hood, M. Dellinger, E. J. Bellefroid, D. S. Jones, A. Hofmann, F. O. Ossa, F. A. Macdonald, C. Wang, T. T. Isson, J. G. Murphy, J. A. Higgins, A. J. West, M. W. Wallace, D. Asael, P. A. E. Pogge von Strandmann, A lithium-isotope perspective on the evolution of carbon and silicon cycles. *Nature* **595**, 394–398 (2021).

98. M. J. Murphy, D. Porcelli, P. A. E. Pogge von Strandmann, C. A. Hirst, L. Kutscher, J. A. Katchinoff, C.-M. Mörth, T. Maximov, P. S. Andersson, Tracing silicate weathering processes in the permafrost-dominated Lena River watershed using lithium isotopes. *Geochim. Cosmochim. Acta* **245**, 154–171 (2019).

99. P. A. E. Pogge von Strandmann, D. N. Schmidt, N. J. Planavsky, G. Wei, C. L. Todd, K.-H. Baumann, Assessing bulk carbonates as archives for seawater Li isotope ratios. *Chem. Geol.* **530**, 119338 (2019).

100. Y. Donnadieu, Y. Goddéris, R. Pierrehumbert, G. Dromart, F. Fluteau, R. Jacob, A GEOCLIM simulation of climatic and biogeochemical consequences of Pangea breakup. *Geochem. Geophys. Geosyst.* **7**, Q11019 (2006).

101. T. M. Lenton, S. J. Daines, B. J. W. Mills, COPSE reloaded: An improved model of biogeochemical cycling over Phanerozoic time. *Earth Sci. Rev.* **178**, 1–28 (2018).

102. J. M. García-Ruiz, S. Beguería, E. Nadal-Romero, J. C. González-Hidalgo, N. Lana-Renault, Y. Sanjuán, A meta-analysis of soil erosion rates across the world. *Geomorphology* **239**, 160–173 (2015).

103. A. Zerboni, A. K. Mishra, C. Placzek, R. Jones, Coupled influence of precipitation and vegetation on millennial-scale erosion rates derived from ^{10}Be. *PLOS ONE* **14**, e0211325 (2019).

104. J. Starke, T. A. Ehlers, M. Schaller, Latitudinal effect of vegetation on erosion rates identified along western South America. *Science* **367**, 1358–1361 (2020).

105. R. A. Berner, GEOCARB III: A revised model of atmospheric CO_2 over Phanerozoic time. *Am. J. Sci.* **301**, 182–204 (2001).

106. J. Bouchez, F. von Blanckenburg, J. A. Schuessler, Modeling novel stable isotope ratios in the weathering zone. *Am. J. Sci.* **313**, 267–308 (2013).

107. J. K. Caves Rugenstein, D. E. Ibarra, F. von Blanckenburg, Neogene cooling driven by land surface reactivity rather than increased weathering fluxes. *Nature* **571**, 99–102 (2019).

108. M. Dellinger, J. Bouchez, J. Gaillardet, L. Faure, J. Moureau, Tracing weathering regimes using the lithium isotope composition of detrital sediments. *Geology* **45**, 411–414 (2017).

109. J. Gaillardet, B. Dupre, P. Louvat, C. J. Allegre, Global silicate weathering and CO_2 consumption rates deduced from the chemistry of large rivers. *Chem. Geol.* **159**, 3–30 (1999).

110. R. L. Rudnick, S. Gao, "Composition of the Continental Crust" in *Treatise on Geochemistry* (Elservier, ed. 2, vol. 4, 2014), pp. 1–51.

111. S. Z. Shen, S. L. Mei, Lopingian (Late Permian) high-resolution conodont biostratigraphy in Iran with comparison to South China zonation. *Geol. J.* **45**, 135–161 (2010).

112. L. F. Gou, Z. D. Jin, P. A. E. P. von Strandmann, G. Li, Y. X. Qu, J. Xiao, L. Deng, A. Galy, Li isotopes in the middle Yellow River: Seasonal variability, sources and fractionation. *Geochim. Cosmochim. Acta* **248**, 88–108 (2019).

113. J. Wang, A. D. Jacobson, H. Zhang, J. Ramezani, B. B. Sageman, M. T. Hurtgen, S. A. Bowring, S.-Z. Shen, Coupled $\delta^{44/40}$Ca, $\delta^{88/86}$Sr, and ^{87}Sr/^{86}Sr geochemistry across the end-Permian mass extinction event. *Geochim. Cosmochim. Acta* **262**, 143–165 (2019).

114. S. Misra, P. N. Froelich, Lithium isotope history of Cenozoic seawater: Changes in silicate weathering and reverse weathering. *Science* **335**, 818–823 (2012).

115. Y. Huh, L.-H. Chan, L. Zhang, J. M. Edmond, Lithium and its isotopes in major world rivers: Implications for weathering and the oceanic budget. *Geochim. Cosmochim. Acta* **62**, 2039–2051 (1998).

116. L. Sauzéat, R. L. Rudnick, C. Chauvel, M. Garçon, M. Tang, New perspectives on the Li isotopic composition of the upper continental crust and its weathering signature. *Earth Planet. Sci. Lett.* **428**, 181–192 (2015).
